# The Cacti Microbiome: Interplay between Habitat-Filtering and Host-Specificity

**DOI:** 10.3389/fmicb.2016.00150

**Published:** 2016-02-12

**Authors:** Citlali Fonseca-García, Devin Coleman-Derr, Etzel Garrido, Axel Visel, Susannah G. Tringe, Laila P. Partida-Martínez

**Affiliations:** ^1^Departamento de Ingeniería Genética, Centro de Investigación y de Estudios AvanzadosIrapuato, Mexico; ^2^Department of Energy Joint Genome InstituteWalnut Creek, CA, USA; ^3^Environmental Genomics and Systems Biology Division, Lawrence Berkeley National LaboratoryBerkeley, CA, USA; ^4^Plant Gene Expression Center, United States Department of Agriculture-Agricultural Research ServiceAlbany, CA, USA; ^5^Molecular Cell Biology, School of Natural Sciences, University of California, MercedMerced, CA, USA

**Keywords:** arid and semi-arid ecosystems, Cactaceae, CAM plants, holobiont, Illumina amplicon sequencing, microbial diversity, microbiomes, plant-microbe interactions

## Abstract

Cactaceae represents one of the most species-rich families of succulent plants native to arid and semi-arid ecosystems, yet the associations Cacti establish with microorganisms and the rules governing microbial community assembly remain poorly understood. We analyzed the composition, diversity, and factors influencing above- and below-ground bacterial, archaeal, and fungal communities associated with two native and sympatric Cacti species: *Myrtillocactus geometrizans* and *Opuntia robusta.* Phylogenetic profiling showed that the composition and assembly of microbial communities associated with Cacti were primarily influenced by the plant compartment; plant species, site, and season played only a minor role. Remarkably, bacterial, and archaeal diversity was higher in the phyllosphere than in the rhizosphere of Cacti, while the opposite was true for fungi. Semi-arid soils exhibited the highest levels of microbial diversity whereas the stem endosphere the lowest. Despite their taxonomic distance, *M. geometrizans* and *O. robusta* shared most microbial taxa in all analyzed compartments. Influence of the plant host did only play a larger role in the fungal communities of the stem endosphere. These results suggest that fungi establish specific interactions with their host plant inside the stem, whereas microbial communities in the other plant compartments may play similar functional roles in these two species. Biochemical and molecular characterization of seed-borne bacteria of Cacti supports the idea that these microbial symbionts may be vertically inherited and could promote plant growth and drought tolerance for the fitness of the Cacti holobiont. We envision this knowledge will help improve and sustain agriculture in arid and semi-arid regions of the world.

## Introduction

Arid and semi-arid regions represent one third of the Earth's land surface area (McGinnies, 1979; Peel et al., 2007). These ecosystems are characterized by their low water availability which restrains biological activity (Noy-Meir, 1973). Plants in these habitats are subjected to many types of abiotic stress including extreme temperature fluctuations, high ultraviolet radiation, low nutrient content soils, and drought. Moreover, climate change studies predict that agriculture in dry lands will be particularly threatened as global temperature increases and rainfall patterns are disturbed (Cline, 2007; Collins et al., 2008).

Cacti (Cactaceae), together with agaves and other xerophytes, represent keystone species in arid and semi-arid ecosystems in the American continent, where they originated around 32.11 million years ago (Hernández-Hernández et al., 2014). Cactaceae are an extremely diverse family of plants; to date, around 2000 species have been identified, distributed from Southwest Canada to Southern Argentina (Bravo Hollis and Sheinvar, 1999). This rich family of succulent plants use the *crassulacean acid metabolism* (CAM) for photosynthesis, a biochemical strategy that allows them to fix carbon dioxide during the night and prevent water loss during the day (Nobel, 2003). This strategy, together with morphological changes such as a thin and superficial root systems (which enable water uptake in moisture-limiting conditions), and succulent bodies (used for water storage), have likely enabled their success in arid and semi-arid regions around the globe (Nobel, 2010).

It is now well-recognized that plants and animals form tight associations with microorganisms resulting in a “holobiont” (i.e., host plus all its microorganisms), which represents an evolutionary unit of selection and biological organization (Rosenberg et al., 2010; Partida-Martínez and Heil, 2011; Bordenstein and Theis, 2015; Vandenkoornhuyse et al., 2015). However, in most plants and animals, we still lack the basic knowledge of who these associated microorganisms are, which factors most influence their association with the host, and what is their role in the fitness of the holobiont. Several studies have recently analyzed the influence of a number of factors on the plant microbiome, such as the host genotype, the plant compartment, the soil and its intrinsic physical and chemical properties, the seasonal variations, the agricultural management, the biogeography of the plant species and the plant development (Caruso et al., 2011; Lundberg et al., 2012; Chaparro et al., 2014; Desgarennes et al., 2014; Kembel et al., 2014; Lebeis, 2014; Maignien et al., 2014; Schlaeppi et al., 2014; Bulgarelli et al., 2015; Coleman-Derr et al., 2016). In Cacti, however, most of these questions remain poorly understood.

Nevertheless, previous studies have shown that both bacteria and fungi are able to densely colonize the rhizoplane of different species, such as *Pachycereus pringlei, Stenocereus thurbei*, and *Opuntia cholla* (Puente et al., 2004a). Indeed, some of these bacterial strains have been characterized and found to be responsible for rock weathering and growth promotion in Cacti developing on rocky cliffs, large rocks, or ancient lava flows (Puente et al., 2004b, 2009b; Lopez et al., 2011). Another study that combined both culture dependent and independent methods showed that *Actinobacteria, Proteobacteria*, and *Acidobacteria* were the most represented Phyla associated with the rhizosphere of *Mammillaria carnea, Opuntia pilifera*, and *Stenocereus stellatus*, with *Actinobacteria, Proteobacteria*, and members of the *Firmicutes* the most frequently recovered by isolation techniques (Aguirre-Garrido et al., 2012). In the case of fungi, reports to date have shown that unidentified fungi formed part of the rhizoplane of Cacti (Puente et al., 2004a), and that mycorrhizal infection improved P and Zn uptake in *Ferocactus acanthodes*, and CO_2_ uptake in both *F. acanthodes* and *Opuntia ficus-indica* (Cui and Nobel, 1992). A wider screening of cultivable stem endophytic fungi in 21 species of Cacti across different sites in Arizona revealed low diversity of these communities and no host specificity, with *Alternaria* sp., *Aureobasidium pullulans* and *Phoma* spp. identified as the most common fungal microbes associated with these Cacti species, though no functional or ecological role for these Cacti-fungi associations was revealed (Suryanarayanan et al., 2005). Altogether, these studies suggest that Cacti form associations with microorganisms which may be important for their adaptation and survival.

The main focus of this research was to generate an holistic baseline of the associations Cacti establish with bacteria, archaea and fungi in six different plant compartments: the bulk and proximal soil (together referred hereafter as the soils), the rhizosphere and the phyllosphere (the episphere), as well as the root and stem interior (the endosphere) through phylogenetic profiling using the 16S rRNA gene (V4 region) for Bacteria and Archaea, and the nuclear ribosomal internal transcribed spacer region (ITS2) for Fungi. We selected two sympatric species belonging to the main subfamilies of Cactaceae: *Myrtillocactus geometrizans* (Cactoideae) and *Opuntia robusta* (Opuntioideae). These species naturally co-occur in Central Mexico and experience two contrasting seasons (dry and rainy) based on yearly precipitation patterns. We evaluate the impact of the plant host, plant compartment, geographic site, and seasonality on the composition of microbial communities associated with Cacti and infer most relevant microbial partners associated with this group of plants. Finally, we characterize seed-borne bacterial isolates to further deepen our understanding of their potential functions for the interaction with their host plant.

## Materials and methods

### Study system and experimental design

**Figure 1:**
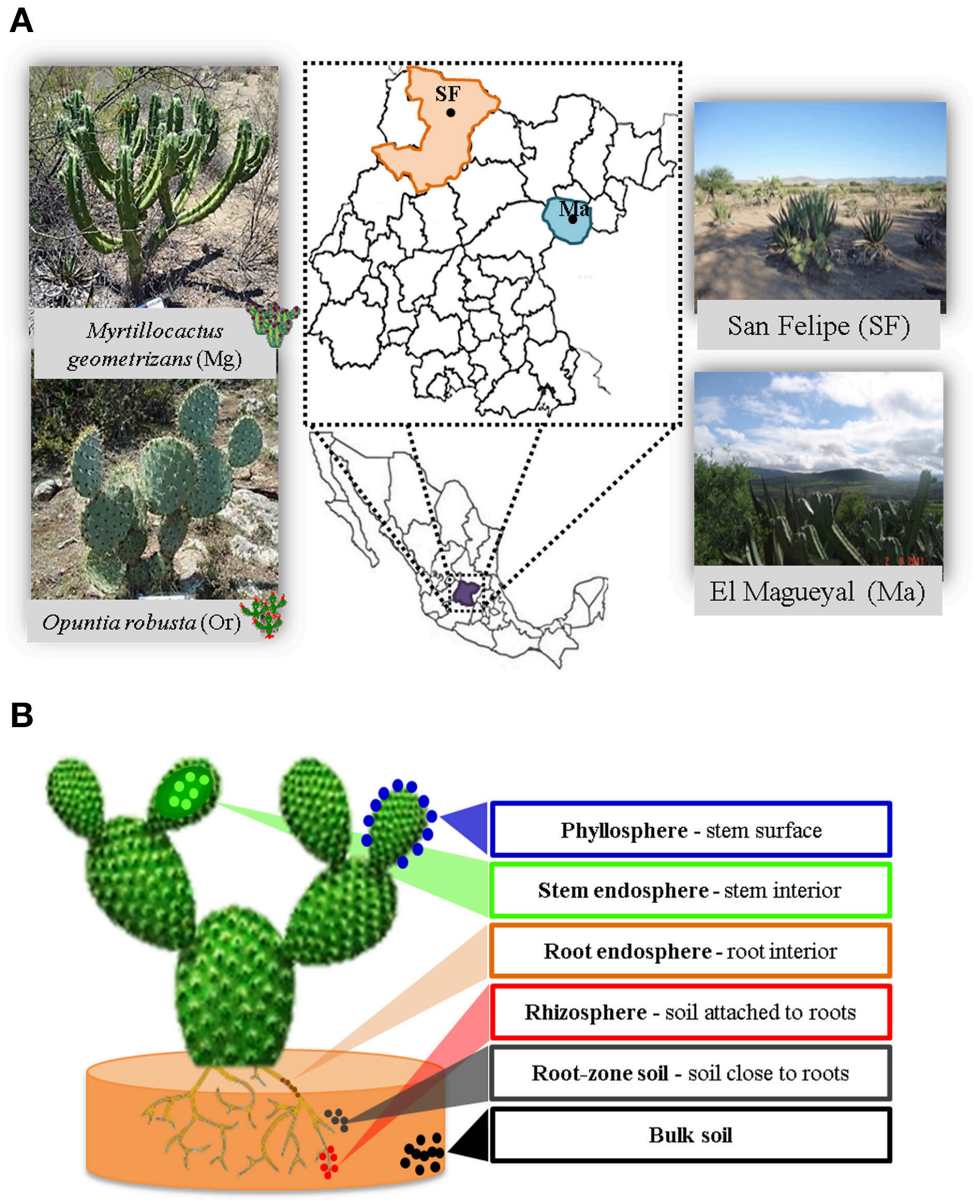
**Experimental design. (A)** Cacti species and sampling sites considered in this study. **(B)** The six plant compartments investigated from each sampled plant.

In order to achieve the goals pursued in this study, it was critical to carefully select the Cacti species to be studied, which should be not endangered and grow in sympatry under natural conditions. The species chosen were *M. geometrizans* (Cactaceae, Subfamily: Cactoideae) and *O. robusta* (Cactaceae, Subfamily: Opuntioideae), both endemic to Mexico (Bravo Hollis and Sheinvar, 1999; Arias et al., 2012) and which have ecological, cultural, and economic relevance (Budinsky et al., 2001; Céspedes et al., 2005; Salazar et al., 2011; Santos-Zea et al., 2011; Vazquez-Cruz et al., 2012; Bolaños-Carrillo et al., 2015; Figure 1A). Two natural populations of *M. geometrizans* and *O. robusta* in the communities of “El Magueyal” and “San Felipe” in Guanajuato, Mexico were identified and selected (Figure 1A). Both sites have semi-arid climate and similar soil characteristics (Table 1). At each site, sampling was done at the end of both the dry and rainy seasons (late May and early October, respectively) in 2012. At each sampling season and site, three healthy, distinct replicate plants of each Cacti species were surveyed (24 plants in total, Table S1). From each plant, six compartments were studied (Figure 1B): the rhizosphere—microorganisms living in the soil firmly attached to the roots; the phyllosphere—microorganisms living on the surface of the stems; the root endosphere—microorganisms living in the interior of roots; the stem endosphere—microorganisms living in the interior of stems; the root-zone soil, which represented loose soil up to 10 cm in proximity to the roots; and the bulk soil which represented soil taken one meter away from the plant, which included soil from the top and up to 10–15 cm depth (Figure S1). The extreme dryness and sandy quality of the soils in which the Cacti specimens were harvested meant in most cases that very little rhizospheric soil remained adhered to the roots upon their removal from the ground. Therefore, we included the root-zone soil samples in the experimental design. Additionally, fruits from both Cacti species were collected in both study sites in late May 2012 to obtain their seeds. For more details, please refer to the Supplementary Material.

**Table 1:** **Soil characteristics, annual mean temperature, and precipitation of the study sites in 2012**.

**Sites**	**El Magueyal (Ma)**	**San Felipe (SF)**
Coordinates	N21°05.106 W100°17.653	N21°39.626 W100°2.959
Altitude (masl)	2175	2089
**ENVIRONMENTAL CONDITIONS IN 2012**		
Annual mean temperature (°C)^a^	18.3	17
Annual mean precipitation (mm)^a^	485	204.5
Precipitation during rainy season (mm)^a^	355	136.5
Precipitation during dry season (mm)^a^	130	68
**SOIL CHARACTERISITICS**		
Texture	Sandy loam	Sandy loam
pH	5.79	6.25
Organic matter (%)	3.97	0.71
Nitrogen (μg g^−1^)	8.76	12.65
Phosphorus (μg g^−1^)	18.11	4.53
Potassium (μg g^−1^)	64.35	251.7

a *Data provided by Comisión Nacional del Agua (CONAGUA)*.

### Sample collection, preparation, and DNA extraction

From each surveyed plant (average height of 82.50 ± 12.47 cm for *M. geometrizans* and 69.50 ± 13.14 cm for *O. robusta*, Table S1), we collected two stems, several roots and two types of soil: the root-zone soil and the bulk soil as defined above (Figure S1). Collected samples were transported to the lab on ice the same day of sampling, prepared the following day and extracted as previously described Desgarennes et al. (2014) to yield total DNA from bulk soil, root-zone soil, rhizosphere, phyllosphere, and root and stem endospheres (Figure 1B) (2 Cacti species × 6 plant compartments × 2 seasons × 2 sites × 3 biological replicates = 144 total DNA samples). For more details, please refer to the Supplementary Material.

### 16S V4 and ITS2 PCR amplification and sequencing of pooled samples

Preliminary analyses performed on the 16S rRNA gene (V6-V8 region) of endophytic Cacti samples using DGGE as reported before (Desgarennes et al., 2014) helped us estimate that intravariation among the three biological replicates from each Cacti species at each site and season was low (Figure S2). Thus, to reduce sequencing costs and make the project feasible, we decided to pool equivalent amounts of DNA from the three biological replicates to yield 48 pooled samples (144 individual samples/3 biological replicates = 48 pooled samples). These samples were further processed using the MiSeq Illumina platform to sequence amplicons of the bacterial and archaeal 16S rRNA (V4 region) and the fungal ITS2 as previously described (Coleman-Derr et al., 2016). Briefly, we used the well-established 515F (5′ –GTGCCAGCMGCCGCGGTAA– 3′) and 816R (5′ –GGACTACHVGGGTWTCTAAT– 3′) primer set, along with peptide nucleic acid (PNA) clamps to reduce chloroplast and mitochondrial contamination as in Lundberg et al. (2013) for 16S amplification of Bacteria and Archaea, and the ITS9F (5′ –GAACGCAGCRAAIIGYGA– 3′) and ITS4R (5′ –TCCTCCGCTTATTGATATGC– 3′) primer sets for ITS2 fungal amplification. Detailed protocols can be found in the Supplementary Material of this article.

### Sequencing data processing

The Illumina raw reads were processed using a custom pipeline developed at the Joint Genome Institute as reported (Coleman-Derr et al., 2016). Quality, contaminant and reproducibility filters yielded 1,311,071 and 2,752,860 high quality reads for the 16S and ITS2 datasets, respectively (Figure S3). Later, clustering assignment at 97 and 95% sequence identity (see Supplementary Material) was applied to render 4012 bacterial and archaeal and 3541 fungal measurable OTUs, respectively (Data Sheet 1). Due to the relatively small sequence length of the amplified fragments, the lowest achievable taxonomic level for both the 16S and ITS2 datasets is Genus. For most downstream analyses, all measurable OTUs were used (as defined in the Supplemental Material, Accession Numbers KU536055 - KU539595). However, for alpha diversity analyses, we accounted for differences in sequencing read depth across plant compartments by randomly subsampling all samples (rarefied) to 275 and 375 reads per sample in the 16S rRNA gene and ITS2 datasets, respectively. By doing so, we were able to compare diversity indexes for all plant compartments for both Cacti species. All generated sequences have been deposited under SRA accession: SRP068631. For more details on the sequence data processing methods, please refer to the Supplementary Material.

### Statistical analyses

In order to identify the principal factors that influence microbial community composition, we used Analysis of Similarity (AnoSim) with 999 permutations, unless otherwise stated, and Non-Metric Multidimensional Scaling analysis (NMDS) on all pair wise Bray–Curtis dissimilarities for both bacterial/archaeal and fungal datasets as previously reported (Desgarennes et al., 2014; Coleman-Derr et al., 2016). The aim of the NMDS ordination method is to display the dissimilarities between the samples (i.e., objects) graphically, that is, the distance between the objects on the plot represent their relative dissimilarities considering the abundance of the OTUs they share. The measure of the goodness of fit of the final NMDS plot is the stress value (*S*), which indicates the match between inter-object distance and dissimilarity. The closer the *S*-value is to zero (ideally less than 0.1), the better the ordination (Manly, 1986; Quinn and Keough, 2002). These analyses were performed in R (R Core Team, 2013) using the function *anosim* of the Vegan package and the function *isoMDS* of the MASS package. Other statistical analyses such as the Kruskal–Wallis and Dunn tests were also performed in R using the functions *kruskal.test* and *dunn.test*, respectively.

The alpha diversity of the microbial communities was estimated by the Shannon (*H*′) index using the function *renyiresult* of the BiodiversityR package and the OTUs distribution was presented in Venn diagrams using the function *draw.quad.venn* of the VennDiagram package also in R. Pareto analyses and graphics were performed using the average relative abundance and relative frequency of each OTU in each community (i.e., sample) across the two Cacti species, where 20% or less of the total number of OTUs accounted for 80% of the accumulated relative abundance of a given community.

### Isolation and biochemical characterization of seed-borne bacteria

In order to investigate the role of microorganisms associated with *M. geometrizans* and *O. robusta*, we focused on the seed-borne bacteria. Seeds were first obtained from the fruits collected in the dry season in 2012 as previously mentioned. For this, seeds were separated from the fruit pulp, washed and allowed to dry. Once dried, seeds were sequentially disinfected under sterile conditions in 70% ethanol for 1 min, 0.55% NaOCl for 20 min and two washes of sterile water. Disinfected seeds were germinated and propagated *in vitro* under axenic conditions as described by Estrada-Luna (1988). When seedlings showed differentiation, that is when stem tissues formed, plant tissues derived from three plant seedlings of each Cacti species were disinfected as described by Desgarennes et al. (2014) and placed in Trypticase Soy Agar (TSA) plates at 28°C for 10 days. Bacterial colonies growing on this medium were further diluted and plated until axenic cultures were obtained. Using microbiological methods (macroscopic and microscopic morphology, growth rate, pH, etc.), 17 strains were classified as distinct and were subsequently identified by amplifying and sequencing the 16S rRNA gene as described before (Desgarennes et al., 2014). These sequences were deposited on NCBI under the Accession Numbers: KT937137-KT937153. Finally, partial or complete 16S rRNA gene sequences from the 17 isolated seed-borne bacterial strains were compared against the measurable bacterial/archaeal OTUs sequences generated using the Illumina platform at 97% sequence similarity by means of the CD-HIT software (Li and Godzik, 2006).

We evaluated known traits of plant growth promotion and drought tolerance in the 17 seed-borne strains *in vitro*. First, we evaluated drought tolerance and exopolysaccharide production as described by Kavamura et al. (2013). Second, we evaluated the growth promotion capacity by direct mechanisms such as: qualitative nitrogen fixation (Desgarennes et al., 2014); quantitative auxin (Indole Acetic Acid, IAA) production (Gordon and Weber, 1951); quantitative phosphate solubilization as described by Nautiyal (1999) and qualitative siderophore production as described by De los Santos-Villalobos et al. (2012). Finally, we evaluated the growth promotion capacity by indirect mechanisms through qualitative ammonia production as described by Cappuccino and Sherman (1992); qualitative cyanuric production as described by Bakker and Schippers (1987) and cellulose production as described by Teather and Wood (1982). For these experiments, bacteria were cultured overnight in Tryptic Soy Broth (TSB, Difco, USA) at 28°C and 150 rpm. Cell density was adjusted to an optical density of 1.0 at 600 nm and the experiments performed by triplicate. Mean values and standard deviation of each experiment were calculated and a heat map using normalized data was generated using R. For more details, please refer to the Supplementary Material.

## Results

### Microbial communities associated with cacti and factors affecting their assembly

**Figure 2:**
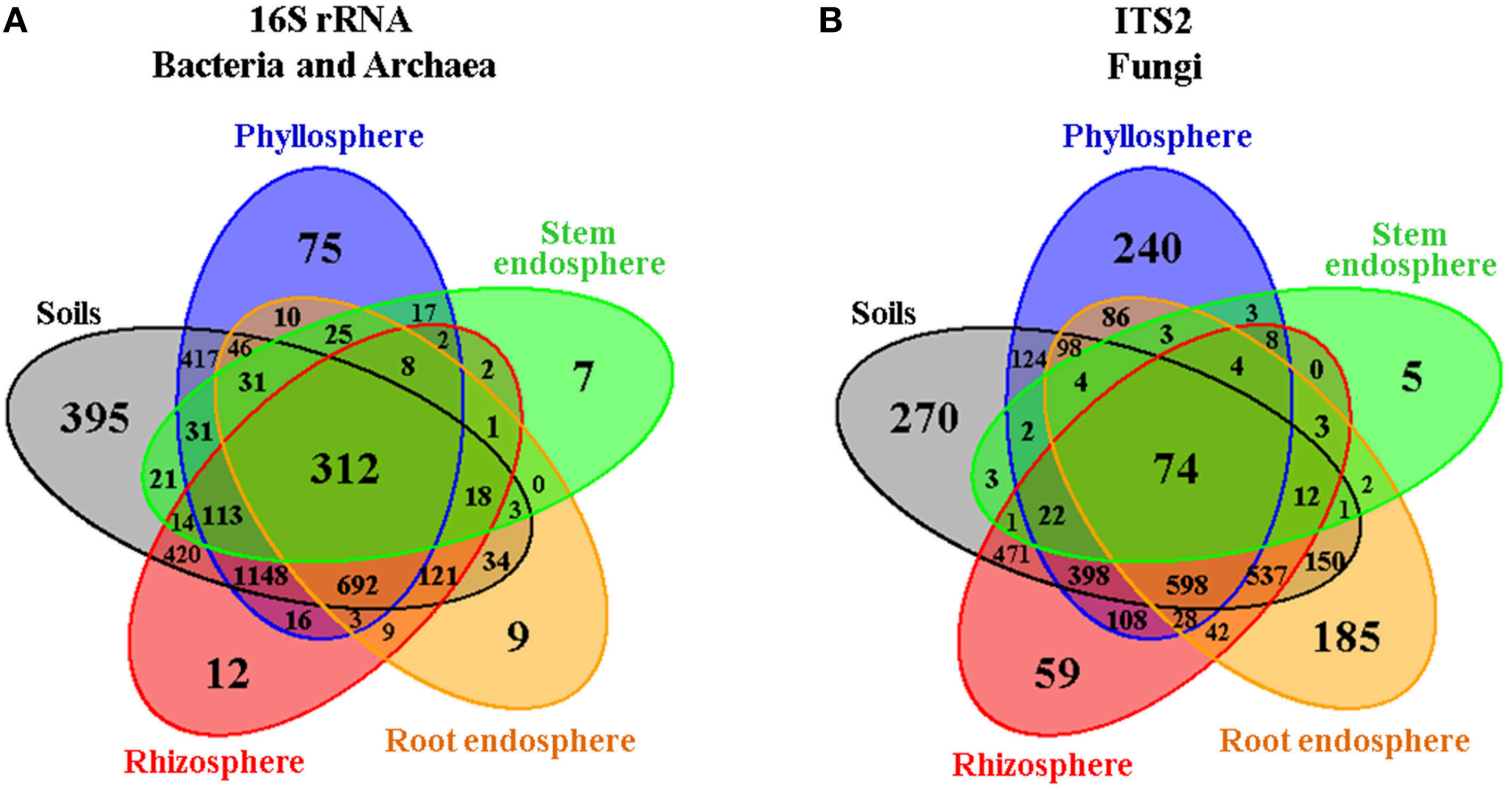
**Venn diagrams showing the distribution of (A) bacterial/archaeal and (B) fungal measurable OTUs associated with Cacti by plant compartment**. The root-zone and bulk soils were grouped together as they showed little differences among them.

In order to gain an overall picture of microbial community structure in Cacti, we first analyzed the distribution of measurable bacterial/archaeal and fungal OTUs by all the factors considered in the experimental design, namely season, site, Cacti species and plant compartment. Comparisons across season, site, and Cacti species showed that 91–92% of identified OTUs were shared across tested conditions in the bacterial/archaeal dataset, while in fungi only 75–80% of the fungal taxa were shared (Figure S4). Venn diagrams displaying OTUs distributed by plant compartment (Figure 2, Figures S5, S6) demonstrated that in both the bacterial/archaeal and fungal dataset, most plant-associated OTUs (i.e., those from the rhizosphere, root endosphere, stem endosphere, and phyllosphere) were also present in the soils. These diagrams also show that a higher proportion of bacterial/archaeal OTUs (7.8%) is shared across the six analyzed plant compartments compared to the fungal dataset (2%). We noted that there were a reduced number of compartment-specific bacterial/archaeal and fungal OTUs, especially in the rhizosphere (0.3 and 1.7%), root endosphere (0.2 and 5.2%), and stem endosphere (0.1 and 0.2%, for bacteria/archaea and fungi respectively), whereas the soils (9.8 and 7.6%) and phyllosphere (1.9 and 6.8%) displayed considerably higher counts. These first results suggested that the microbial communities associated with Cacti were possibly more influenced by the plant compartment than by any of the other factors evaluated.

**Figure 3:**
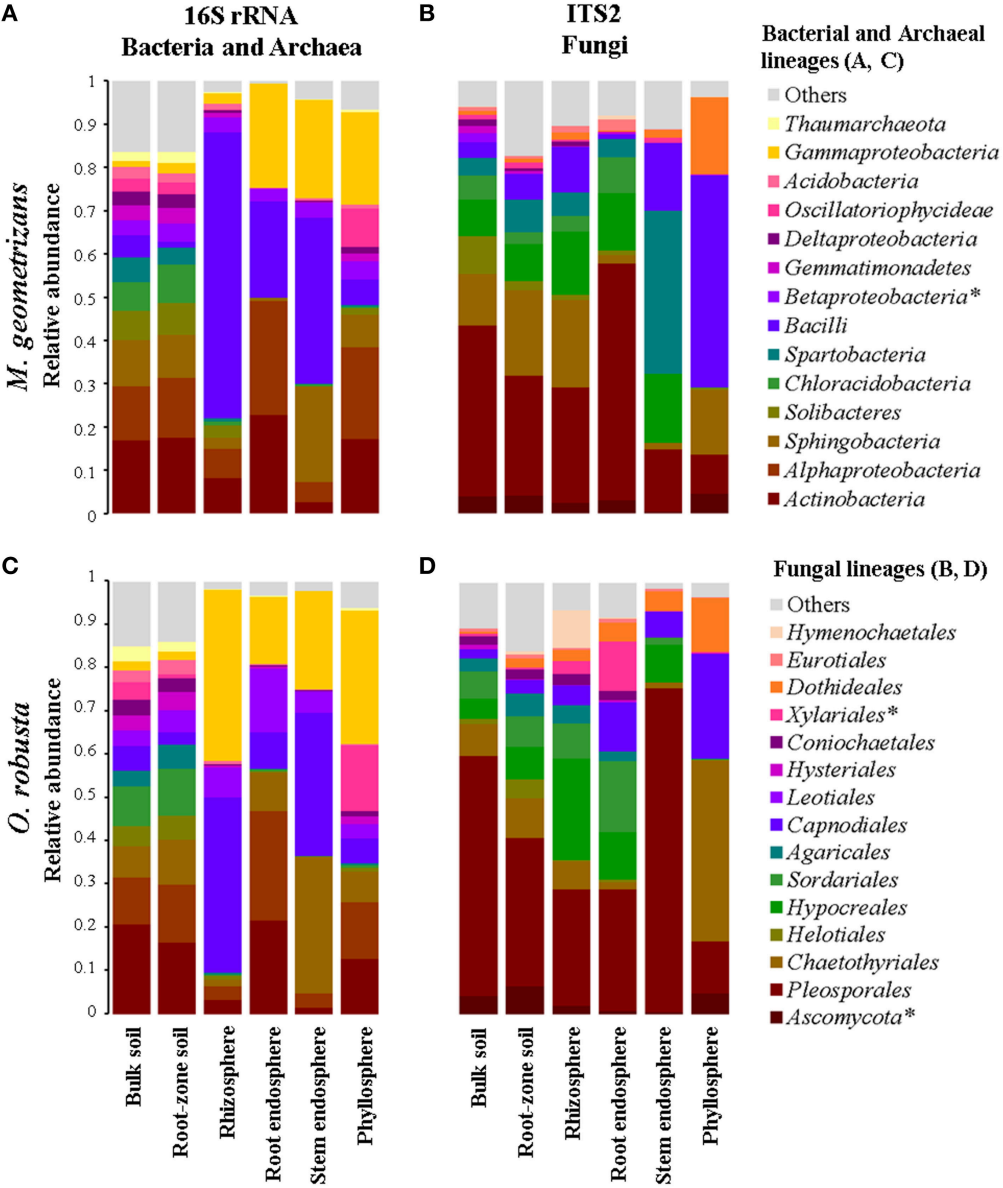
**Relative abundance plots of the microbial communities associated with *M. geometrizans* (A,B) and *O. robusta* (C,D) by plant compartment. (A,C) Class-level composition of the bacterial/archaeal communities and (B,D) Order-level composition of the fungal communities**. The relative abundance of the bacterial/archaeal classes and the fungal orders marked with asterisks were the only ones that did not significantly change among plant compartments in both Cacti species after Kruskal–Wallis test with *P* ≤ 0.05.

Overall, we identified 76 bacterial and two archaeal classes belonging to 32 phyla in all investigated samples, although only 13 bacterial and one archaeal classes were the most prevalent (Figures 3A,C). OTUs belonging to the bacterial phyla *Proteobacteria, Firmicutes, Actinobacteria, Acidobacteria*, and *Bacteroidetes* comprised more than 85% of the relative abundance in each of the four plant-associated communities of *M. geometrizans* and *O. robusta* (rhizosphere, root endosphere, stem endosphere, and phyllosphere). Sequences belonging to the archaeal class *Thaumarchaeota* genus *Nitrososphaera* were uncommon in the plant-associated communities (0.6% average relative abundance), but relatively abundant in the soils (representing on average 3.5% of the community composition). All these bacterial and archaeal classes, with the exception of the *Betaproteobacteria*, displayed significant variations in their relative abundance among the six compartments in both Cacti species (Kruskal–Wallis tests, *P* = 0.05, Tables S2, S3).

In the case of fungi, we detected 17 fungal classes belonging to 6 phyla in our dataset, with the phylum *Ascomycota* being the most abundant (more than 90% of the total relative abundance) in the plant-associated communities. These communities were principally composed of members of the ascomycotan orders *Pleosporales, Chaetothyriales, Capnodiales, Dothideales*, and *Hypocreales*, and of the basidiomycotan *Agaricales* and *Hymenochaetales* (Figures 3B,D). All these fungal orders, except for the *Xylariales* and sequences identified only as *Ascomycota*, displayed significant variations in their relative abundance among the six compartments in both Cacti species (Kruskal–Wallis tests, *P* = 0.05, Tables S2, S3). Sequences belonging to arbuscular mycorrhizal fungi of the genera *Entrophospora* and *Glomus* (phylum *Glomeromycota*) represented less than 0.5% of the relative abundance in the root-zone soil and rhizosphere of both Cacti species, suggesting low or absent levels of plant colonization.

The microbial composition of the bulk and root-zone soil samples were similar across plant species, but the plant-associated communities (rhizosphere, root endosphere, stem endosphere, and phyllosphere) were clearly different from the soils, and were also distinct from each other and between plant host species (Figure 3). In the soils, we identified 395 and 270 specific bacterial/archaeal and fungal taxa (Figures 2A,B), which represented 117 and 131 genera, respectively. Nevertheless, these diverse taxa only accounted for about 1.8% of the total relative abundance in these soil samples, suggesting that the most abundant soil borne microorganisms were also able to colonize one or more plant-associated compartments of both Cacti species.

AnoSim results considering all biotic and abiotic factors included in the experimental design indicated that plant compartment was the principal driver of both bacterial/archaeal and fungal community composition, explaining 62.8 and 66% of the dissimilarities respectively (Table 2). Interestingly, the plant species, season, and site were only significant in their combination with plant compartment, meaning that their role is also a function of the plant compartment. This interaction was slightly higher in the fungal than in the bacterial/archaeal communities. Because of the strong effect of plant compartment on microbial communities, we performed separate AnoSim analyses for each compartment (Table 2) and for two plant-associated samples subgroups: above- and below-ground, as well as epi- and endo-phytic communities plus the soils (Table S4). These analyses not only confirmed that the plant compartment was the most important factor affecting microbial assembly, but also that the plant species influenced bacterial and fungal stem endosphere, bacterial root endosphere and fungal phyllosphere. On the other hand, the site played only a role in the fungal communities of the rhizosphere, bulk and root-zone soils.

**Table 2:** **Analysis of similarity (AnoSim) using the Bray–Curtis dissimilarity matrix across all Cacti samples considering all factors and their interactions (only significant factors are displayed *P* ≤ 0.057)**.

	**Factor**	**16S rRNA Bacteria and Archaea**		**ITS2 Fungi**	
		**AnoSim R**	***P***	**AnoSim R**	***P***
**Global (all 48 samples)**	^a^Plant compartment_6, 48, 999_	0.6285	0.001	0.6600	0.001
	^b^Plant compartment_6_ by Species_2, 8, 999_	0.6285	0.001	0.6600	0.001
	^b^Plant compartment_6_ by Season_2, 8, 999_	0.6285	0.001	0.6600	0.001
	^b^Plant compartment_6_ by Site_2, 8, 999_	0.6285	0.001	0.6600	0.001
	^b^Species_2_ by Plant compartment_6, 24, 999_	−0.0022	0.005	0.0142	0.001
	^b^Season_2_ by Plant compartment_6, 24, 999_			0.0004	0.016
	^b^Site_2_ by Plant compartment_6, 24, 999_	−0.0080	0.016	0.0459	0.001
**Phyllosphere**	^a^Species_2, 8, 999_			0.2708	0.057
**Stem endosphere**	^a^Species_2, 8∕7, 999_	0.7604	0.027	0.3148	0.056
	^b^Species_2_ by Season_2, 4∕3, 576∕144_	0.7604	0.057	0.3148	0.041
	^b^Species_2_ by Site_2, 4, 576_	0.7604	0.050		
**Root endosphere**	^a^Species_2, 7, 999_	0.4630	0.030		
	^b^Species_2_ by Season_2, 3, 144_	0.4630	0.056		
	^b^Season_2_ by Site_2, 4, 576_			0.2188	0.054
**Rhizosphere**	^a^Site_2, 8, 999_			0.6979	0.031
	^b^Site_2_ by Season_2, 4, 576_			0.6979	0.054
**Root-zone soil**	^a^Site_2, 8, 999_			0.4792	0.027
	^b^Site_2_ by Species_2, 4, 576_			0.4792	0.039
**Bulk soil**	^a^Site_2, 8, 999_			0.8646	0.027
	^b^Site_2_ by Season_2, 4, 576_			0.8646	0.038

a *Subscript numbers separated by comas indicate for each factor: the number of levels, number of total replicates, and permutations employed in each AnoSim test*.

b *Subscript numbers indicate for the first factor the number of levels and for the second factor, the subscript numbers separated by comas: the number of levels, number of total replicates, and permutations employed in each AnoSim test*.

*In particular cases, the diagonal separates the bacteria/archaea (left) and fungi (right) data*.

**Figure 4:**
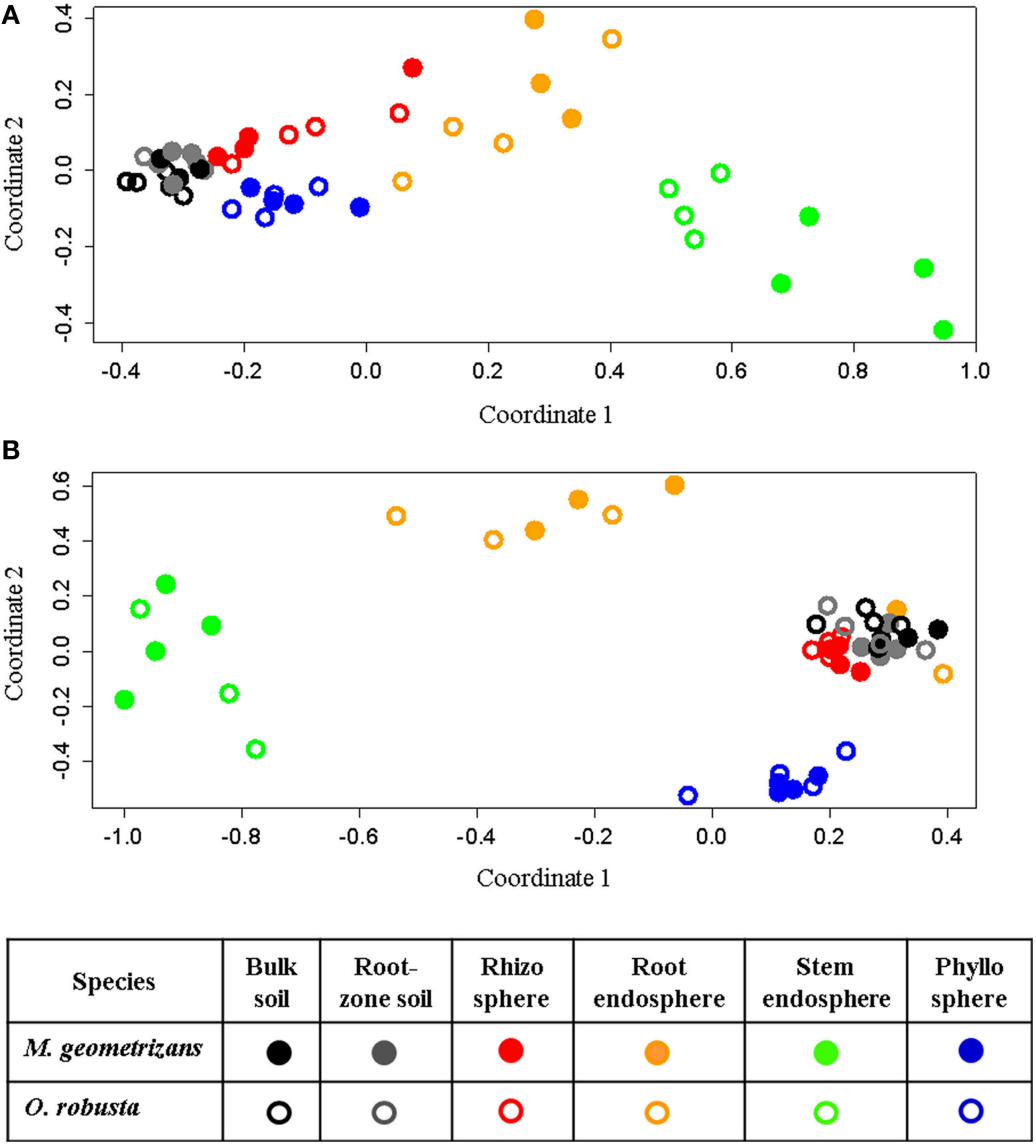
**Non-metric multidimensional scaling (NMDS) plots for Bray–Curtis distances of the (A) bacterial/archaeal (*S* = 0.07) and (B) fungal (*S* = 0.11) communities associated with *M. geometrizans* (shaded symbols) and *O. robusta* (empty symbols)**.

NMDS analyses based on Bray–Curtis dissimilarity matrixes corroborated these AnoSim results. These ordination plots showed that in the 16S dataset (Figure 4A), clusters were formed based on plant compartment. Specifically, Coordinate 1 (x-axis) separated samples from the soils (bulk and root-zone soils) followed by the epiphytic samples (rhizosphere and phyllosphere) in the left (all four outside the plant) from the endophytic samples (root and stem endosphere) in the right, whereas the Coordinate 2 (y-axis) showed in its center the soil samples, in the upper part the below-ground communities (rhizosphere and root endosphere), and in the lower part the above-ground assemblages (phyllosphere and stem endosphere). This plot also reveals that only clusters for the stem endophytic samples formed distinct subclusters based on the host plant (Figure 4A), and the rhizosphere, phyllosphere, root endosphere, and stem endosphere form a joint continuum radiating away from the soil samples. On the other hand, the NMDS plot depicting the fungal communities shows four clearly distinct clusters grouping samples from the stem endosphere in the left; the root endosphere in the upper-center; the phyllosphere in the lower-right, and a mixed cluster in the middle-right integrated mainly by rhizospheric and soil samples, plus two rainy root endophytic samples obtained from the San Francisco site (Figure 4B). Analyses performed only on the endosphere revealed that the plant host species had an influence in both microbial communities of the stem endosphere, as samples from each Cacti species cluster together (Figure S7).

### Alpha diversity of the microbial communities associated with cacti

**Figure 5:**
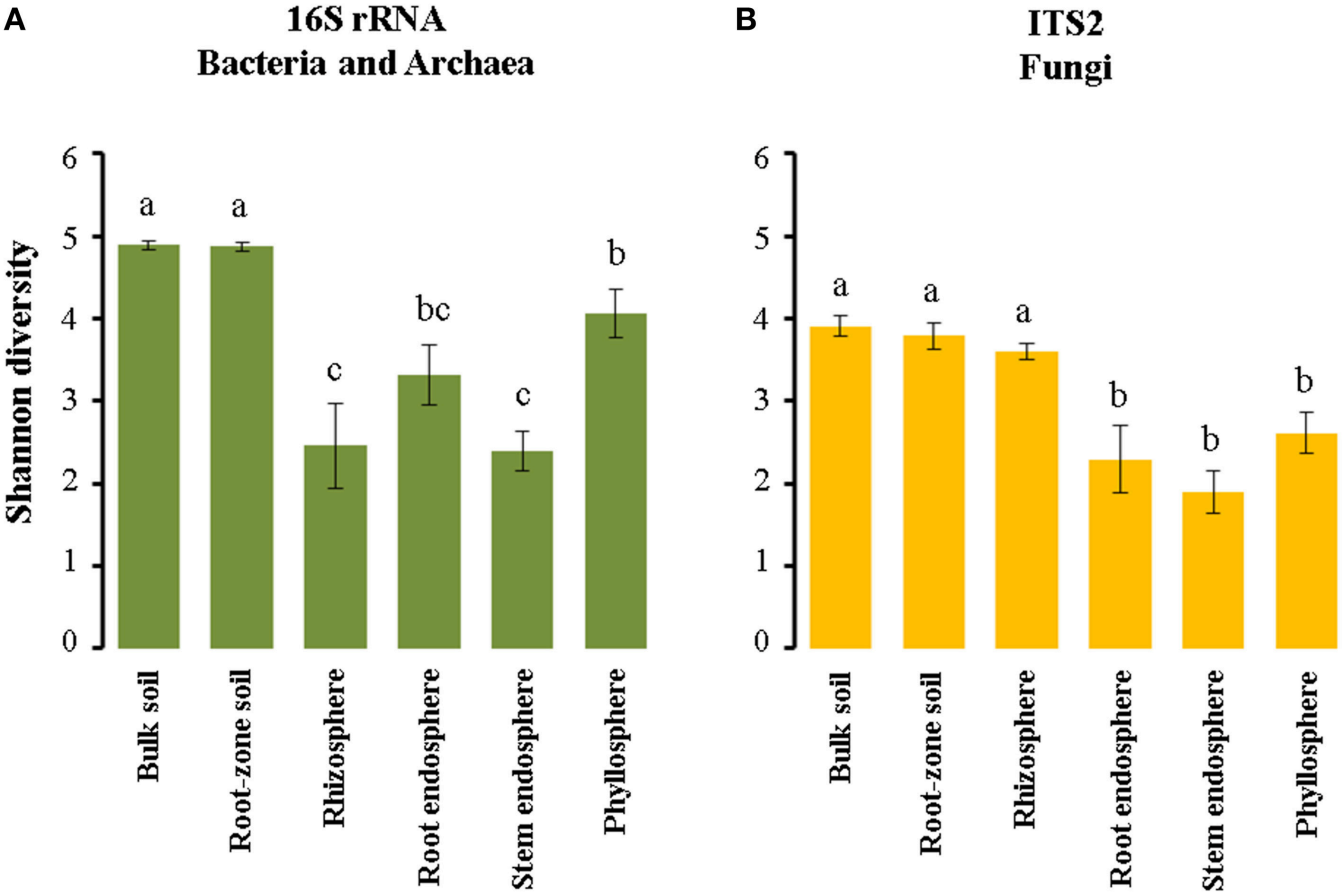
**Estimated Shannon (*H*′) diversity index in the (A) bacterial/archaeal and (B) fungal communities associated with both *M. geometrizans* and *O. robusta***. Different letters on the top of the bars represent significant differences among plant compartments after Kruskal–Wallis test and Dunn test with *P* ≤ 0.05.

We used the Shannon index in order to compare microbial diversity in the communities associated with Cacti. Our analyses showed that there were not significant differences in alpha diversity between *M. geometrizans* and *O. robusta*, neither between the dry and rainy seasons nor between sites (Table S5). There were however significant differences between the six plant compartments for both the bacterial/archaeal and fungal communities in both species of Cacti (Figure 5, Table S6). In the bacterial/archaeal communities, alpha diversity of the bulk and root-zone soil communities was significantly higher relative to the diversity in the epiphytic and endophytic communities associated with both species. Remarkably, we observed that alpha diversity in the phyllosphere was significantly higher than in the rhizosphere, while the root endosphere exhibited slightly higher alpha diversity than the diversity of the stem endosphere (Figure 5A). In the case of fungi, the Shannon index showed that the fungal alpha diversity in the soils and the rhizosphere was significantly higher relative to the diversities in the root and stem endospheres and phyllosphere of both species (Figure 5B).

### Habitat-filtering vs. host-specificity in the microbial communities associated with sympatric cacti

**Figure 6:**
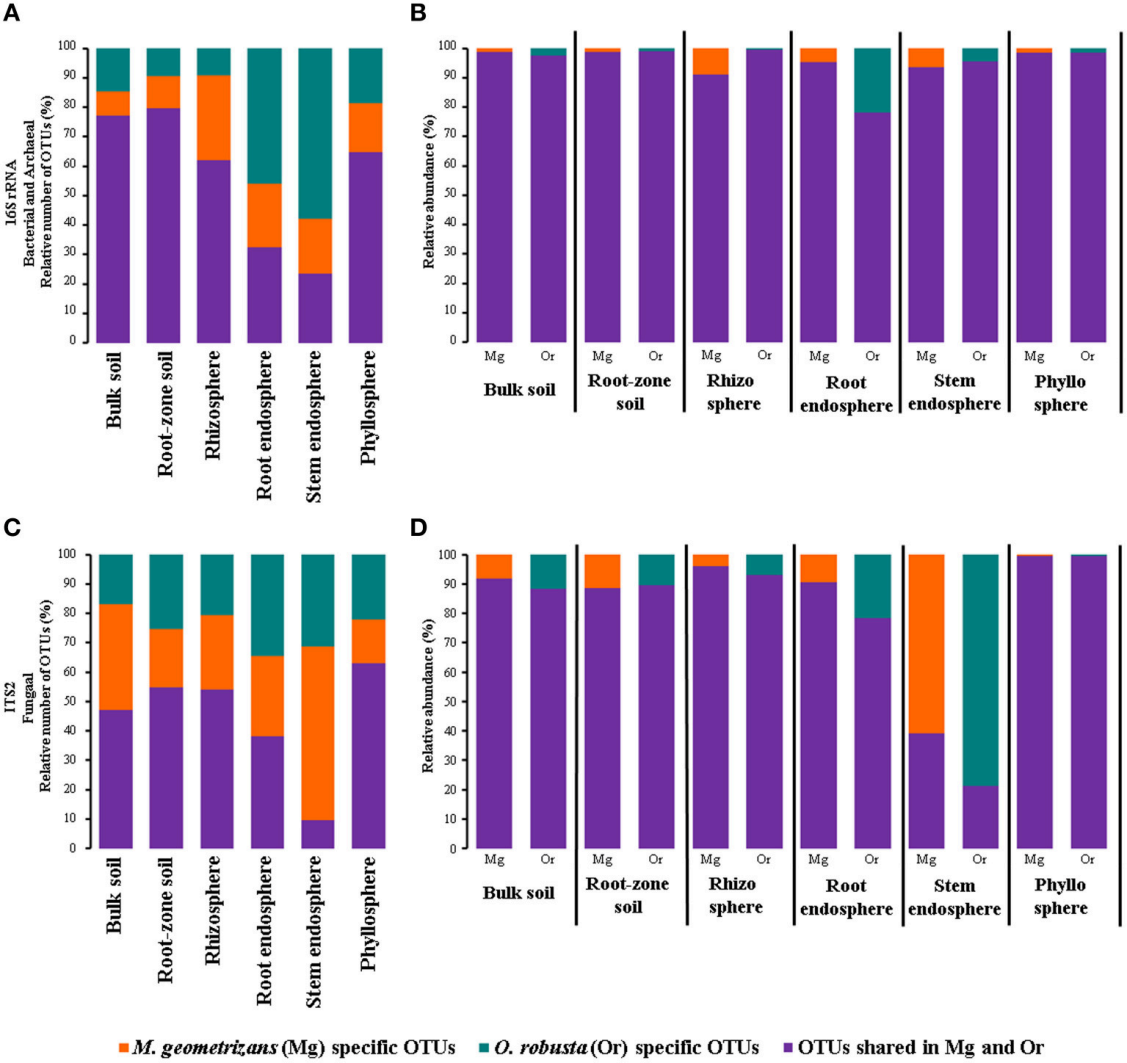
**Relative number (A,C) and abundance (B,D) of species-specific and shared bacterial/archaeal (A,B) and fungal (C,D) measurable OTUs by plant compartment in *M. geometrizans* and *O. robusta***.

One of the key questions motivating this research was to determine the specific influence of the habitat (environment) and the plant species in the composition of the microbiota, specially in harsh natural environments such as the semi-arid and arid ecosystems where Cacti species flourish (Habitat-filtering vs. Host-specificity). To answer this, we first determined the number and relative abundance of shared and species-specific microbial OTUs in each compartment associated with sympatric *M. geometrizans* and *O. robusta* (Figure 6). These analyses showed that in the bacterial/archaeal communities (Figure 6A), the two Cacti species shared the highest number of OTUs in the soils (ca. 78%) and a larger number of OTUs in the rhizosphere and phyllosphere (ca. 63% of the total OTUs associated with each community), than they did in the root and stem endosphere (33 and 23%, respectively). Nevertheless, shared bacterial/archaeal OTUs represented the majority of reads across the six communities (80–98% of the relative abundance in each case; Figure 6B). In fungi (Figure 6C), the proportion of shared taxa among Cacti species was lower than for the bacterial/archaeal dataset and varied considerably among compartments, being highest in the phyllosphere (63% shared OTUs from total) and lowest in the stem endosphere with only 9.5%. However, the contribution of these shared taxa to the total abundance composition was generally high, representing more than 80% in all communities except for the stem endosphere (21–39% of the relative abundance) (Figure 6D). These results reinforce the idea that the host plant played a role in the conformation of the fungal stem endophytic communities.

**Figure 7:**
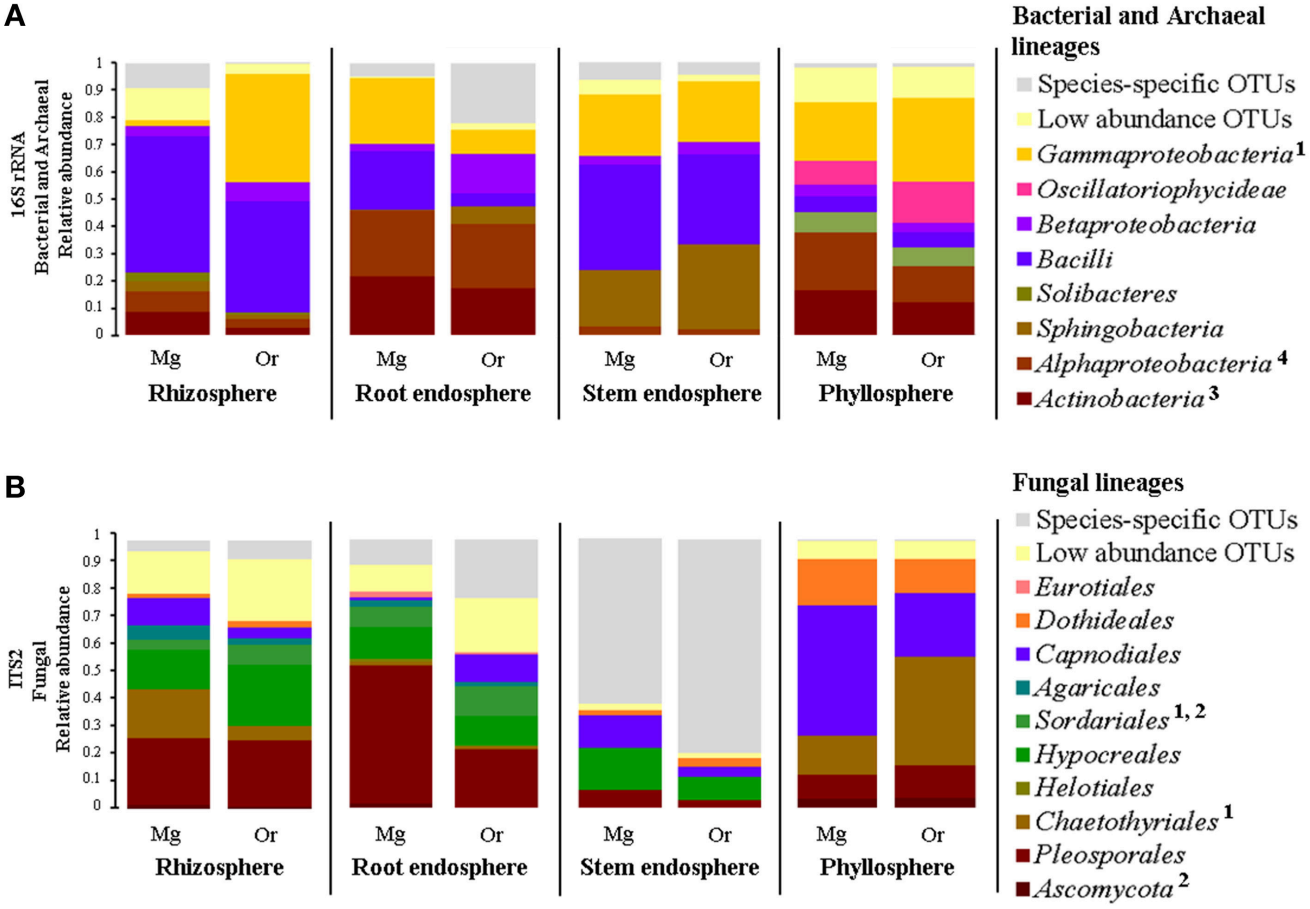
**Taxonomic distribution and relative abundance of shared (A) bacterial/archaeal and (B) fungal measurable OTUs by Cacti species and plant compartment**. The bacterial/archaeal classes and fungal orders marked with numeric superscripts represent statistically significant differences across the two Cacti species after Kruskal–Wallis test with *P* ≤ 0.05 within the following compartments: ^1^rhizosphere, ^2^root endosphere, ^3^stem endosphere and ^4^phyllosphere. Mg, *M. geometrizans*; Or, *O. robusta.*

**Figure 8:**
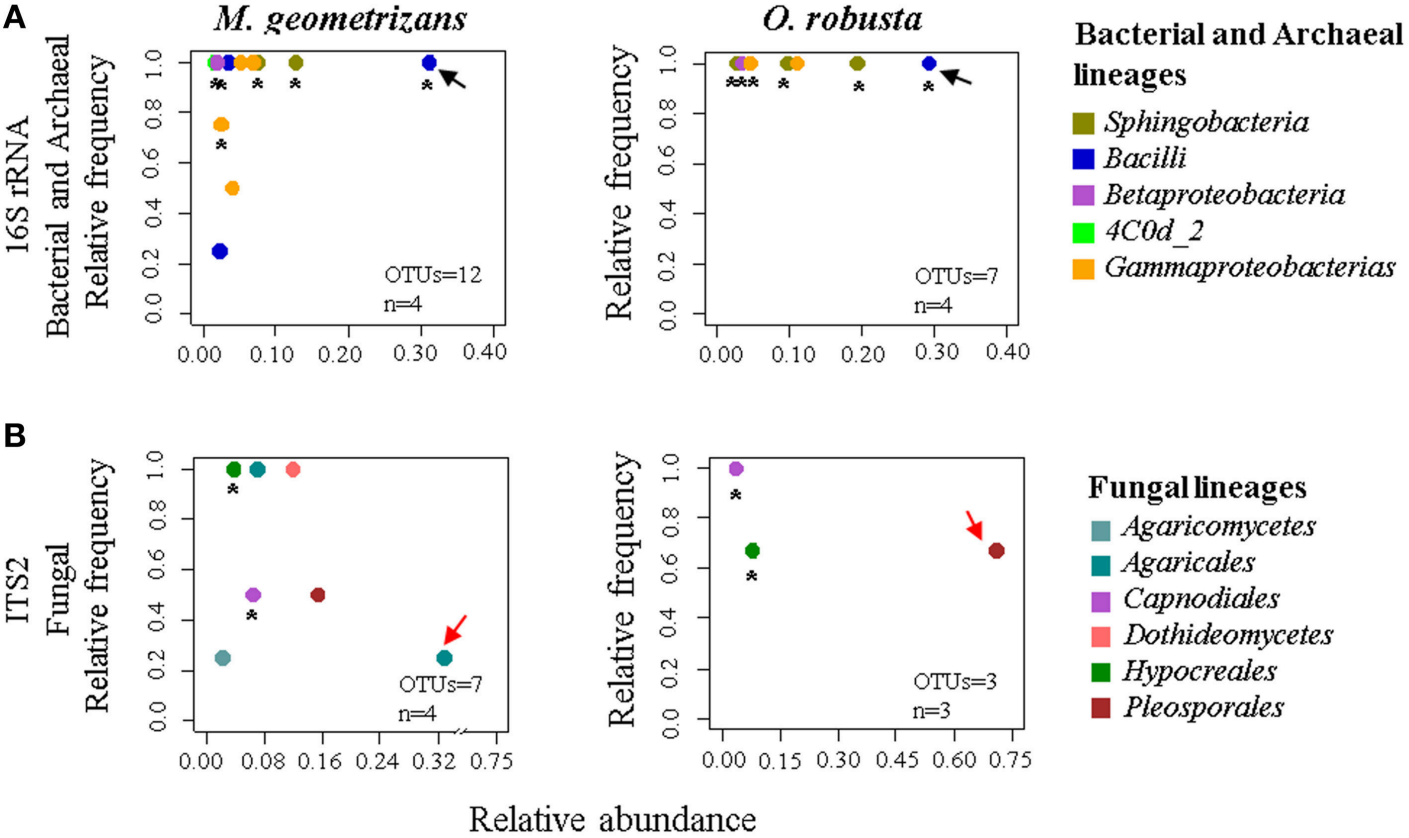
**Pareto analysis of the microbial taxa identified in the stem endosphere of Cacti, where 20% or less of the total number of OTUs account for 80% of the accumulated relative abundance**. Relative frequency vs. relative abundance of **(A)** bacterial/archaeal and **(B)** fungal Pareto taxa in the stem endosphere. Asterisks indicate shared Pareto OTUs. Black arrows in **(A)** indicate OTU 19–*Bacillus* (*Bacilli*) in the bacterial and archaeal plots. Red arrows in **(B)** indicate the fungal species-specific OTUs [in *M. geometrizans* OTU 339–*Henningsomyces* (*Agaricales*), in *O. robusta* OTU 103–*Prathoda* (*Pleosporales*)]. The number of OTUs and the number of samples (n) are indicated in the bottom right corner of each plot.

Based on these results, we were keen in identifying relevant shared microbial taxa associated with both Cacti species at each compartment, while further investigating the influence of plant species in the structure of the microbial assemblages. We combined two analyses to achieve this aim. First, we determined the taxonomic assignment of shared microbial taxa in each compartment and compared their relative abundance among plant species (Figures 7A,B, Table S7). Second, we performed Pareto analyses, where 20% or less of the total number of OTUs accounts for 80% of the accumulated relative abundance of a given community (Figures 8A,B; Figures S8, S9; Data Sheets 2, 3). These analyses revealed that *Bacilli* (mainly a single OTU, OTU 7) dominated the rhizosphere of the two Cacti species; members of the *Gammaproteobacteria* and of the cyanobacterial class *Oscillatoriophycideae* were indicative of the Cacti phyllosphere; *Actinobacteria* and *Alphaproteobacteria* were indicative of the root endosphere (Figures 7A, 8A), while another *Bacilli* (OTU 19) together with taxa related to *Sphingobacteria* were important members of the stem endosphere in these two Cacti species (Figure 8A, OTU 19 indicated by black arrows, Table S8). In the fungal communities, Pareto analyses showed that members of the orders *Hypocreales, Sordariales* (mainly OTU 8), as well as OTUs only identified at the phylum level as *Ascomycota*, were indicative of the rhizosphere of Cacti; members of the *Capnodiales, Dothideales, Chaetothyriales*, and *Pleosporales* dominated the phyllosphere of the investigated Cacti plants. Notably, these fungal taxa were identified in all Cacti plants sampled, although *O. robusta* had a reduced number of OTUs compared to *M. geometrizans*; *Pleosporales, Hypocreales*, and *Sordariales* were indicative of the root endosphere in Cacti (Figure S9). *Capnodiales* (OTU 2–*Cladosporium*) and *Hypocreales* (OTU 320–*Beauveria*) were common to the stem endosphere of both Cacti species, despite the fact that this compartment displayed the highest host specificity (Figure 8B). Pareto analysis of the fungal stem endosphere community also revealed that the *Agaricales* (mainly from the genera *Henningsomyces*, OTU 339) were present in *M. geometrizans* while *Pleosporales* (mainly the genera *Prathoda*, OTU 103) was mainly found in *O. robusta* (Figure 8B, Table S8). Notwithstanding the high similarity in relative abundance composition at the class- and order- level for bacteria/archaea and fungi in most compartments (Figures 7A,B, respectively), Pareto plots of the plant-associated microbial communities revealed differences in the number of Pareto OTUs, their identity and their relative abundance, suggesting that the plant species plays a tuning role in microbial composition assembly (Figures 8A,B; Figures S8, S9).

### *In vitro* biochemical characterization of seed-borne bacteria of cacti

We isolated 17 seed-borne bacterial strains in TSA media. From these, two strains were associated with seeds of *M. geometrizans* and 15 with seeds of *O. robusta* (Table 3). Some of these isolated strains were detected in the 16S dataset in both *M. geometrizans* and *O. robusta*, although they were isolated only from one of the host species. In particular, the strain *Bacillus pumilus* L14 (resembling OTU 19 at 97% sequence identity), isolated from seeds of *O. robusta*, represents around 30% of the total bacterial/archaeal community associated with *M. geometrizans* and *O. robusta*, especially in the stem endosphere. This result suggests that the strain *B. pumilus* L14, a likely vertically transmitted bacterium, can play an important role in the adult host plants.

**Table 3:** **Identification of seed-borne bacterial strains associated with *M. geometrizans* and *O. robusta***.

**Strain**	**Isolated from…**	**Putative OTU**	**Overall relative abundance (%)**		**Plant compartment with the highest relative abundance**	
			***M. geometrizans***	***O. robusta***	***M. geometrizans***	***O. robusta***
*Bacillus pumilus* L14	*O. robusta*	19	30.91	29.30	Stem endosphere	Stem endosphere
*Bacillus pumilus* L1	*O. robusta*					
*Bacillus pumilus* L3	*O. robusta*					
*Staphylococcus hominis* L12	*O. robusta*	99	3.60	2.50	Stem endosphere	Stem endosphere
*Psychrobacillus psychrodurans* L4	*O. robusta*	–				
*Psychrobacillus psychrodurans* L5	*O. robusta*	–				
*Psychrobacillus psychrodurans* L6	*O. robusta*	–				
*Psychrobacillus psychrodurans* L7	*O. robusta*	–				
*Psychrobacillus psychrodurans* L8	*O. robusta*	–				
*Psychrobacillus psychrodurans* L9	*O. robusta*	–				
*Psychrobacillus psychrodurans* L2	*O. robusta*	–				
*Psychrobacillus psychrodurans* L10	*O. robusta*	–				
*Psychrobacillus psychrodurans* L11	*O. robusta*	–				
*Psychrobacillus psychrodurans* L13	*O. robusta*	–				
*Aarococcus terreus* L15	*O. robusta*	–				
*Leclercia adecarboxylata* L16	*M. geometrizans*	4	13.63	18.53	Stem endosphere	Rhizosphere
*Nocardiopsis prasina* L17	*M. geometrizans*	680	0.01	0.11	Phyllosphere	Stem endosphere

**Figure 9:**
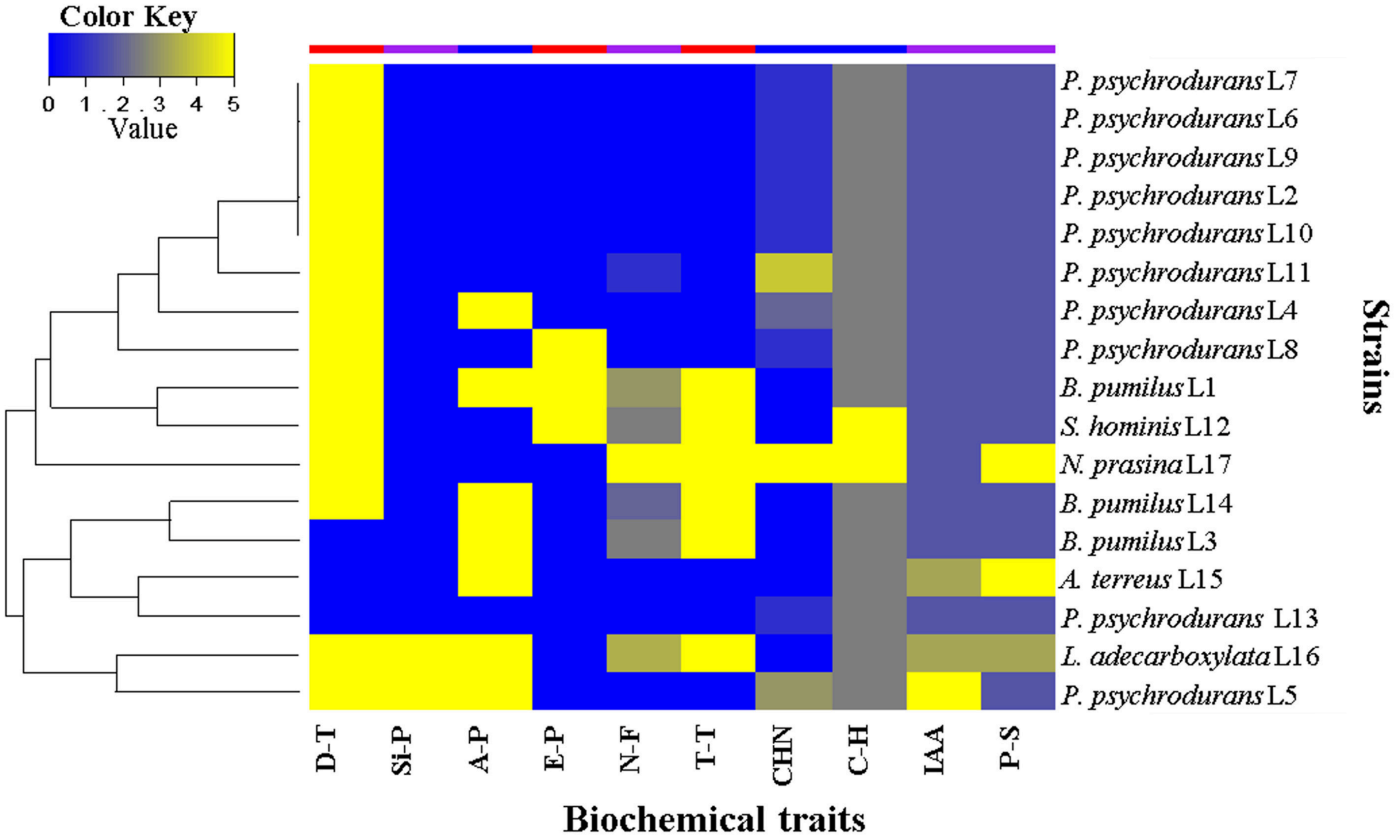
**Heat map of the biochemical traits displayed by seed-borne bacterial strains isolated from *M. geometrizans* and *O. robusta***. D-T, drought tolerance; Si-P, siderophore production; A-P, ammonia production; E-P, exopolysaccharide production; N-F, nitrogen fixation; T-T, high temperature tolerance; HCN, hydrogen cyanide production; C-H, carboxymethylcellulose hydrolysis; IAA, indole acetic acid production (1–30 μgIAA/ml); and P-S, phosphate solubilization (1–300 μgPO_4_/ml). Values range from 0 to 5, where 0 represents absence or null activity and 5 represents presence or the highest quantitative value of activity.

In order to investigate the role of the 17 isolated bacterial strains, we characterized some plant growth promotion traits *in vitro*. The results are summarized in Figure 9. We first evaluated the drought tolerance by measurements of bacterial growth under reduced water availability and at high temperature, together with the capacity to produce exopolysaccharides. These experiments showed that 80% of the strains were able to grow under reduced water availability, whereas 45% were able to grow at 40°C. Exopolysaccharides were produced only by the strains *Staphylococcus hominis* L12, *B. pumilus* L14, and *B. pumilus* L3. We then evaluated the growth promotion capacity by direct mechanisms, where 47% of the strains were able to fix nitrogen; all the strains produced above 1.0 μg/mL of IAA (only *Psychrobacillus psychrodurans* L5 produced c.21 μg/mL); all were able to solubilize phosphate, where around 24% of them solubilized above 100 μg/mL (Table S9), and the strains *P. psychrodurans* L5 and *Leclercia adecarboxilata* L16 were able to produce siderophores in the CAS medium. Finally, we evaluated the growth promotion capacity by indirect mechanisms, where around 40% of the strains were able to produce ammonia; cyanuric production was detected in around 60% of the strains, and 100% of the strain were able to hydrolyze carboxymethylcellulose as carbon source (only *S. hominis* L12 and *Nocardiopsis prasina* L17 produced hydrolysis halo). These results highlight that the seed-borne bacterial strains we isolated do possess plant growth promoting traits that may contribute to the plant holobiont fitness.

## Discussion

### The biotic factor plant compartment drives the microbial community composition in cacti

It is now acknowledged that the assembly of plant-associated microbial communities is influenced by environmental filters, such as soil characteristics, seasonal changes, host genotype, and stochastic forces (Grayston et al., 1998; Costa et al., 2006; Caruso et al., 2011; Lundberg et al., 2012; Vorholt, 2012; Chaparro et al., 2014; Kembel et al., 2014; Lebeis, 2014; Maignien et al., 2014; Schlaeppi et al., 2014; Bulgarelli et al., 2015; Edwards et al., 2015; Makhalanyane et al., 2015; Massimo et al., 2015; Zarraonaindia et al., 2015; Coleman-Derr et al., 2016). In this study, we analyzed the bacterial/archaeal and fungal communities associated with six compartments of two native and sympatric Cacti species under comparable abiotic factors such as site and season. Our results showed that the plant species, the biogeography of the plant species, and the season did not strongly influence microbial composition assembly. However, there were clear differences among microbial communities by plant compartment (the rhizosphere, root, and stem endosphere, phyllosphere and the bulk and root-zone soils), revealing this biotic factor as the main driver of selection. The biogeography (site) of the sampled Cacti species did not have a significant effect probably due to the similar soil characteristics and environmental conditions between the sites of “El Magueyal” and “San Felipe” in 2012 (Table 1), which are located 130 km apart. This pattern was also observed in *Agave salmiana*, a native *Agave* species co-occurring in the same populations as the Cacti plants investigated here, using both DGGE-fingerprinting and Illumina sequencing as previously reported (Desgarennes et al., 2014; Coleman-Derr et al., 2016). Recent studies in model and agricultural crop plants have shown that the plant compartment (micro-habitat) explains most of the variance in microbial composition, although the majority of them have focused on the below-ground bacterial/archaeal communities, given less attention to above-ground assemblages and fungi (Singh et al., 2009; Khidir et al., 2010; Lundberg et al., 2012; Jin et al., 2014; Edwards et al., 2015; Massimo et al., 2015; Zarraonaindia et al., 2015). Our results in Cacti, which encompassed three important microbial groups: Bacteria, Archaea, and Fungi, and both external and internal as well as above- and below-ground communities, support the notion that plant compartment is the major selective force shaping plant-microbe interactions in arid and semi-arid habitats, congruent with the findings reported for Agaves (Desgarennes et al., 2014; Coleman-Derr et al., 2016).

### The complex above-ground microbial diversity

Microbial community diversity has been directly related to system functionality, in particular to the efficiency of resource utilization (Hooper et al., 2012). This relationship suggests that losses in microbial diversity may directly impact desert biomes, where functional guilds with little or no redundancy (e.g., nitrogen assimilation) may be the most sensitive (Hilton et al., 2013; Philippot et al., 2013; Coleman-Derr et al., 2016). In Cacti, as has been reported in others studies with succulent and non-succulent plants (Singh et al., 2009; Köberl et al., 2011; Lundberg et al., 2012; Marasco et al., 2012; Desgarennes et al., 2014; Makhalanyane et al., 2015; Zarraonaindia et al., 2015), our data showed that the soil possess the highest taxonomic microbial diversity considering both bacteria/archaea and fungi, while the stem endosphere had the lowest. Remarkably, the bacterial/archaeal communities in the phyllosphere of Cacti showed higher diversity than that of the rhizosphere, despite the fact that communities in the phyllosphere are directly exposed to high UV radiation and higher temperature gradients. This result resembles the ones reported for native *A. salmiana* and *A. deserti*, in which bacterial/archaeal diversity in the phyllosphere was slightly higher or equivalent to the diversity in the rhizosphere (Desgarennes et al., 2014; Coleman-Derr et al., 2016). To our knowledge, this high alpha diversity in the bacterial/archaeal communities in the phyllosphere has only been reported for Agaves and now in Cacti. We hypothesize that this higher alpha diversity could be related to the presence of the cyanobacterial class *Oscillatoriophycideae*, found mainly in the form of the genera *Microcoleus* and *Phormidium*. These cyanobacterial genera were also present in the phyllosphere of native *Agave* species (Coleman-Derr et al., 2016) and a number of reports have shown that Cyanobacteria, such as *Microcoleus* spp., represent pioneer species in aerial and extreme habitats such as Biological Soil Crusts of deserts (Gorbushina, 2007; Nunes da Rocha et al., 2015; Zhang et al., 2015; Čapková et al., 2016), due to their autotrophy, tolerance to dehydration, capacity to fix nitrogen, and form biofilms that function as water and nutrient reservoirs, which protect the embedded cells from adverse environmental conditions and UV radiation (Omoregie et al., 2004; Bolhuis et al., 2010; Rajeev et al., 2013; Makhalanyane et al., 2015). Thus, it is possible that the cyanobacterial species identified here may provide a significant input of nitrogen and a comfortable environment with both moisture and abiotic stress protection for the proliferation of other bacterial/archaeal and fungal groups under arid conditions. This hypothesis warrants further investigation.

On the other hand, the fungal communities exhibited similar diversity levels in the soils and rhizosphere of Cacti, suggesting low levels of selection exerted by the latter plant-associated compartment. This pattern is similar to what has been reported for fungal communities of both native and cultivated Agaves (Coleman-Derr et al., 2016). The fungal endophytic communities and the phyllosphere had lower diversity in Cacti, but a certain degree of host-specificity. Few studies have investigated epiphytic and/or endophytic fungi associated with plants under arid conditions. The few existing reports had relied on culture-dependent methods and had been performed on non-succulent woody plants (Massimo et al., 2015) and diverse Cacti species (Suryanarayanan et al., 2005), both in the Sonoran desert. In these studies, members of the phylum *Ascomycota* from the classes *Dothideomycetes, Eurotiomycetes*, and *Sordariomycetes* were the dominant community members, as was found in our study. At the genus level, *Alternaria, Cladosporium*, and *Aureobasidium* were previously reported in Cacti and were also present in *M. geometrizans* and *O. robusta*, suggesting these fungal species could be well-adapted to the endosphere of Cacti (generalist), despite the geographic distance between the Sonoran desert and Central Mexico. Notably, the most prominent fungal endophytes of non-succulent desert woody plants were *Preussia, Phoma, Botryosphaeria, Penicillium, Aspergillus*, and *Chaetomium* (Massimo et al., 2015), while in our study *Prathoda* in *O. robusta* and the basidiomycotan genus *Henningsomyces* in *M. geometrizans* were more abundant, suggesting that communities in the above-ground endosphere of Cacti are composed by both host-specific strains together with some generalists.

It remains an open question to elucidate how stem endophytic microbes colonize this compartment and how inheritable they are. Cacti often reproduce in nature by vegetative reproduction, which enhances the probability of inheriting the internal microbiota from the parent. It will be interesting to test if these putative host-specific strains are only capable of colonizing their selected host, or if this observed specificity is due to stochastic events that have been preserved and maintained in local populations thanks to this mode of reproduction.

### The microbiome of cacti

Our taxonomic distribution analysis of bacteria, archaea, and fungi between *M. geometrizans* and *O. robusta* showed that these sympatric Cacti species shared a considerable number of bacterial/archaeal and fungal OTUs in the rhizosphere and phyllosphere, representing in each case around 90% of the microbial composition of these communities. Nevertheless, Pareto analyses revealed that communities were secondary influenced by the plant host, as Pareto microbial partners differed in number, identity, abundance and prevalence across the two species. Considering that the Opuntioideae and Cactoideae subfamilies diverged 18 and 16 million years ago respectively (Hernández-Hernández et al., 2014), it is striking that the epiphytic communities are so similar among them, as shown here. We contrasted our results to those obtain for Agaves (Coleman-Derr et al., 2016), which are phylogenetically unrelated to Cacti, but share the CAM metabolism and often the same ecological niche (Nobel, 2010). These comparisons suggest that habitat exerts a stronger selective force than host identity and its evolutionary history, as most abundant microbial taxa identified in each plant compartment were shared across these two groups of plants as presented here and previously (Desgarennes et al., 2014; Coleman-Derr et al., 2016). These results suggest that habitat-filtering exerts a larger force than plant species in shaping microbial assemblies, which is in accordance with studies made on other plant systems such as *A. thaliana* and some of its relatives (Lundberg et al., 2012; Schlaeppi et al., 2014; Bulgarelli et al., 2015), maize (Peiffer et al., 2013), and rice (Edwards et al., 2015). In model plants and agricultural crops, host specificity in the rhizosphere has been mainly attributed to radical exudates and volatile compounds produced by the plant host (Wieland et al., 2001; Yang et al., 2001; Haichar et al., 2008; Hunter et al., 2010; Weinert et al., 2010; Hardoim et al., 2011; Kavamura et al., 2013; Schlaeppi et al., 2014; Bulgarelli et al., 2015; Müller et al., 2015; Sapkota et al., 2015). However, these metabolic differences have not been quantified. Our results in Cacti reinforce the idea that the harsh environmental conditions that Cacti and Agaves face promote similar plant-microbe associations, which may contribute to their adaptation and survival (Desgarennes et al., 2014; Coleman-Derr et al., 2016). It is still necessary to evaluate if these shared microbial taxa are also present in other sympatric succulent and non-succulent desert plants as well as to investigate if they truly represent the same taxa (strain-level differences not captured by present OTU resolution) or if speciation processes are occuring to a significant degree.

Contrary to the epiphytic microbial communities, the assemblies in the endosphere did show signs of host specificity. This was more dramatic in the fungal communities of the stem endosphere. Until now, the studies performed in plants of arid systems have not reported fungal specificity by the host in the stem endosphere (Suryanarayanan et al., 2005; Massimo et al., 2015). However, the fungal community of the stem endosphere of *M. geometrizans* and *O. robusta* revealed host-specific microbes with high relative abundance suggesting a possible functional specificity, which could be related to specific physiological characteristics of each host plant. To date, the *M. geometrizans* specific fungal genera *Henningsomyces* has been reported as ligninolytic degrader (Freitag and Morrell, 1992), while no studies on *Prathoda* are available to infer their likely role in *O. robusta*. Thus, an avenue for continued investigation is the targeted isolation of these stem endophytic fungal genera to test their specificity, heritability and likely functional role *in planta*.

Finally, our collection of 17 seed-borne bacteria from Cacti and their biochemical characterization suggest that these microorganisms possess traits related to drought tolerance and growth promotion. This result resembles the one reported for endophytic bacteria from seeds of *P. pringlei* that can improve the development of cactus seedlings growing in pulverized rock (Puente et al., 2009a). Currently, the genomes of most of these likely vertically transmitted bacterial strains are being sequenced. This genetic information, together with controlled green-house experiments, will allow us to determine if these bacterial strains are mutualistic symbionts of Cacti and their role in plant fitness.

In summary, the present study provides a holistic perspective of the composition, diversity and forces shaping bacterial/archaeal and fungal communities in Cacti and suggests avenues to further understand plant-microbe interactions in arid and semi-arid environments.

## Author contributions

LPP-M, AV, and SGT–Planned and designed research; CF-G and LPP-M–Selected sampling sites, collected, and prepared samples of *M. geometrizans* and *O. robusta*; DC-D–Prepared libraries and processed Illumina amplicon sequencing data; CF-G and EG–performed statistical analyses; CF-G and LPP-M analyzed the data and wrote the paper; All authors read and approved the final manuscript.

## Funding

LPP-M acknowledges Consejo Nacional de Ciencia y Tecnología in Mexico (CONACyT), which supported this project with two grants: CB-2010-01-151007 and INFR-2012-01-197799 as well as the support from the JGI Community Science Program (CSP) under Contract No. DE-AC02-05CH11231.

### Conflict of interest statement

The authors declare that the research was conducted in the absence of any commercial or financial relationships that could be construed as a potential conflict of interest.

## References

[B1] Aguirre-Garrido J. F. Montiel-Lugo D. Hernández-Rodríguez C. Torres-Cortes G. Millán V. Toro N.. (2012). Bacterial community structure in the rhizosphere of three cactus species from semi-arid highlands in central Mexico. Antonie Van Leeuwenhoek 101, 891–904. 10.1007/s10482-012-9705-3 22307841

[B2] Arias S. Gamma S. Guzmán L. (2012). Fascículo 95. Cactaceae, in Flora del Valle de Tehuacán-Cuicatlán, eds Medina Lemos R. Sánchez Ken J. G. García Mendoza A. Arias Montes S. (Mexico, DF: SNIB-CONABIO and Instituto de Biología UNAM), 1–246.

[B3] Bakker A. W. Schippers B. (1987). Microbial cyanide production in the rhizosphere in relation to potato yield reduction and *Pseudomonas* spp.-mediated plant growth-stimulation. Soil Biol. Biochem. 19, 451–457. 10.1016/0038-0717(87)90037-X

[B4] Bolaños-Carrillo M. A. Ventura-Gallegos J. L. Saldivar-Jiménez A. D. Zentella-Dehesa A. Martínez-Vázquez M. (2015). Effect of sterols isolated from *Myrtillocactus geometrizans* on growth inhibition of colon and breast cancer cells. Evid. Based Compl. Altern. Med. 2015:589350. 10.1155/2015/589350 26113867 PMC4465765

[B5] Bolhuis H. Severin I. Confurius-Guns V. Wollenzien U. Stal L. (2010). Horizontal transfer of the nitrogen fixation gene cluster in the cyanobacterium *Microcoleus chthonoplastes*. ISME J. 4, 121–130. 10.1038/ismej.2009.99 19741736

[B6] Bordenstein S. R. Theis K. R. (2015). Host Biology in light of the microbiome: ten principles of holobionts and hologenomes. PLoS ONE 13:e1002226. 10.1371/journal.pbio.1002226 26284777 PMC4540581

[B7] Bravo Hollis H. Sheinvar L. (1999). El Interesante Mundo de las Cactáceas, 2nd Edn. Mexico: CONACYT-Fondo de Cultura Económica.

[B8] Budinsky A. Wolfram R. Oguogho A. Efthimiou Y. Stamatopoulos Y. Sinzinger H. (2001). Regular ingestion of *Opuntia robusta* lowers oxidation injury. Prostaglandins Leukot. Essent. Fatty Acids 65, 45–50. 10.1054/plef.2001.0287 11487308

[B9] Bulgarelli D. Garrido-Oter R. Münch P. C. Weiman A. Dröge J. Pan Y.. (2015). Structure and function of the bacterial root microbiota in wild and domesticated barley. Cell Host Microbe 17, 392–403. 10.1016/j.chom.2015.01.011 25732064 PMC4362959

[B10] Čapková K. Hauer T. Řeháková K. Doležal J. (2016). Some like it high! Phylogenetic diversity of high-elevation cyanobacterial community from biological soil crusts of western Himalaya. Microb. Ecol. 71, 113–123. 10.1007/s00248-015-0694-4 26552394

[B11] Cappuccino J. C. Sherman N. (1992). Microbiology: A Laboratory Manual, 3rd Edn. New York, NY: Benjamin/Cummings Publishing Company.

[B12] Caruso T. Chan Y. Lacap D. C. Lau M. C. Y. McKay C. P. Pointing S. B. (2011). Stochastic and deterministic processes interact in the assembly of desert microbial communities on a global scale. ISME J. 5, 1406–1413. 10.1038/ismej.2011.21 21368908 PMC3160679

[B13] Céspedes C. L. Salazar J. R. Martínez M. Aranda E. (2005). Insect growth regulatory effects of some extracts and sterols from *Myrtillocactus geometrizans* (Cactaceae) against *Spodoptera frugiperda* and *Tenebrio molitor*. Phytochemistry 66, 2481–2493. 10.1016/j.phytochem.2005.07.010 16122768

[B14] Chaparro J. M. Badri D. V. Vivanco J. M. (2014). Rhizosphere microbiome assemblage is affected by plant development. ISME J. 8, 790–803. 10.1038/ismej.2013.196 24196324 PMC3960538

[B15] Cline W. R. (2007). Global Warming and Agriculture: Impact Estimates by Country. Washington, DC: Center for Global Development and Peterson Institute for International Economics.

[B16] Coleman-Derr D. Desgarennes D. Fonseca-Garcia C. Gross S. Clingenpeel S. Woyke T.. (2016). Plant compartment and biogeography affect microbiome composition in cultivated and native *Agave* species. New Phytol. 209, 798–811. 10.1111/nph.13697 26467257 PMC5057366

[B17] Collins S. L. Sinsabaugh R. L. Crenshaw C. Green L. Porras-Alfaro A. Stursova M. (2008). Pulse dynamics and microbial processes in aridland ecosystems. J. Ecol. 96, 413–420. 10.1111/j.1365-2745.2008.01362.x

[B18] Costa R. Götz M. Mrotzek N. Lottmann J. Berg G. Smalla K. (2006). Effects of site and plant species on rhizosphere community structure as revealed by molecular analysis of microbial guilds. FEMS Microbiol. Ecol. 56, 236–249. 10.1111/j.1574-6941.2005.00026.x 16629753

[B19] Cui M. Nobel P. S. (1992). Nutrient status, water uptake and gas exchange for three desert succulents infected with mycorrhizal fungi. New Phytol. 122, 643–649. 10.1111/j.1469-8137.1992.tb00092.x

[B20] De los Santos-Villalobos S. Barrera-Galicia G. C. Miranda-Salcedo M. A. Peña-Cabriales J. J. (2012). Burkholderia cepacia XXVI siderophore with biocontrol capacity against Colletotrichum gloeosporioides. World J. Microbiol. Biotechnol. 28, 2615–2623. 10.1007/s11274-012-1071-9 22806187

[B21] Desgarennes D. Garrido E. Torres-Gomez M. J. Peña-Cabriales J. J. Partida-Martinez L. P. (2014). Diazotrophic potential among bacterial communities associated with wild and cultivated *Agave* species. FEMS Microbiol. Ecol. 90, 844–857. 10.1111/1574-6941.12438 25314594

[B22] Edwards J. Johnson C. Santos-Medellín C. Lurie E. Podishetty N. K. Bhatnagar S.. (2015). Structure, variation, and assembly of the root-associated microbiomes of rice. Proc. Natl. Acad. Sci. U.S.A. E911–E920. 10.1073/pnas.1414592112 25605935 PMC4345613

[B23] Estrada-Luna A. A. (1988). Producción de Brotes e Injertación in vitro de seis Especies de Nopal (Opuntia spp.) Originarias del Altiplano Potosino-Zacatecano. Texcoco: Colegio de Posgraduados.

[B24] Freitag M. Morrell J. (1992). Decolorization of the polymeric dye poly r-478 by wood-inhabiting fungi. Can. J. Microbiol. 38, 811–822. 10.1139/m92-133

[B25] Gorbushina A. A. (2007). Life on the rocks. Environ. Microbiol. 9, 1613–1631. 10.1111/j.1462-2920.2007.01301.x 17564597

[B26] Gordon A. S. Weber R. P. (1951). Colorimetric estimation of indole acetic acid. Plant Physiol. 26, 192–195. 10.1104/pp.26.1.192 16654351 PMC437633

[B27] Grayston S. J. Wang S. Campbell C. D. Edwards A. C. (1998). Selective influence of plant species on microbial diversity in the rhizosphere. Soil Biol. Biochem. 30, 369–378. 10.1016/S0038-0717(97)00124-7

[B28] Haichar F. Z. Marol C. Berge O. Rangel-Castro J. I. Prosser J. I. Balesdent J.. (2008). Plant host habitat and root exudates shape soil bacterial community structure. ISME J. 2, 1221–1230. 10.1038/ismej.2008.80 18754043

[B29] Hardoim P. R. Andreote F. D. Reinhold-Hurek B. Sessitsch A. van Overbeek L. S. van Elsas J. D. (2011). Rice root-associated bacteria: insights into community structures across 10 cultivars. FEMS Microbiol. Ecol. 77, 154–164. 10.1111/j.1574-6941.2011.01092.x 21426364 PMC4339037

[B30] Hernández-Hernández T. Brown J. W. Schlumpberger B. O. Eguiarte L. E. Magallón S. (2014). Beyond aridification: multiple explanations for the elevated diversification of cacti in the New World Succulent Biome. New Phytol. 202, 1382–1397. 10.1111/nph.12752 24611540

[B31] Hilton S. Bennett A. J. Keane G. Bending G. D. Chandler D. Stobart R.. (2013). Impact of shortened crop rotation of oilseed rape on soil and rhizosphere microbial diversity in relation to yield decline. PLoS ONE 8:e59859. 10.1371/journal.pone.0059859 23573215 PMC3613410

[B32] Hooper D. U. Adair E. C. Cardinale B. J. Byrnes J. E. K. Hungate B. A. Matulich K. L.. (2012). A global synthesis reveals biodiversity loss as a major driver of ecosystem change. Nature 486, 105–108. 10.1038/nature11118 22678289

[B33] Hunter P. J. Hand P. Pink D. Whipps J. M. Bending G. D. (2010). Both leaf properties and microbe-microbe interactions influence within-species variation in bacterial population diversity and structure in the lettuce (lactuca species) phyllosphere. Appl. Environ. Microbiol. 76, 8117–8125. 10.1128/AEM.01321-10 20952648 PMC3008232

[B34] Jin H. Yang X. Y. Yan Z. Q. Liu Q. Li X. Z. Chen J. X.. (2014). Characterization of rhizosphere and endophytic bacterial communities from leaves, stems and roots of medicinal *Stellera chamaejasme* L. Syst. Appl. Microbiol. 37, 376–385. 10.1016/j.syapm.2014.05.001 24958606

[B35] Kavamura V. N. Santos S. N. Da Silva J. L. Parma M. M. Ávila L. A. Visconti A.. (2013). Screening of Brazilian cacti rhizobacteria for plant growth promotion under drought. Microbiol. Res. 168, 183–191. 10.1016/j.micres.2012.12.002 23279812

[B36] Kembel S. W. O'Connor T. K. Arnold H. K. Hubbell S. P. Wright S. J. Green J. (2014). Relationships between phyllosphere bacterial communities and plant functional traits in a neotropical forest. Proc. Natl. Acad. Sci. U.S.A. 111, 13715–13720. 10.1073/pnas.1216057111 25225376 PMC4183302

[B37] Khidir H. H. Eudy D. M. Porras-Alfaro A. Herrera J. Natvig D. O. Sinsabaugh R. L. (2010). A general suite of fungal endophytes dominate the roots of two dominant grasses in a semiarid grassland. J. Arid Environ. 74, 35–42. 10.1016/j.jaridenv.2009.07.014

[B38] Köberl M. Müller H. Ramadan E. M. Berg G. (2011). Desert farming benefits from microbial potential in arid soils and promotes diversity and plant health. PLoS ONE 6:e24452. 10.1371/journal.pone.0024452 21912695 PMC3166316

[B39] Lebeis S. L. (2014). The potential for give and take in plant – microbiome relationships. Front. Plant Sci. 5:287. 10.3389/fpls.2014.00287 24999348 PMC4064451

[B40] Li W. Godzik A. (2006). Cd-hit: a fast program for clustering and comparing large sets of protein or nucleotide sequences. Bioinformatics 22, 1658–1659. 10.1093/bioinformatics/btl158 16731699

[B41] Lopez B. R. Bashan Y. Bacilio M. (2011). Endophytic bacteria of *Mammillaria fraileana*, an endemic rock-colonizing cactus of the southern Sonoran Desert. Arch. Microbiol. 193, 527–541. 10.1007/s00203-011-0695-8 21445557

[B42] Lundberg D. S. Lebeis S. L. Herrera Paredes S. Yourstone S. Gehring J. Malfatti S.. (2012). Defining the core *Arabidopsis thaliana* root microbiome. Nature 488, 86–90. 10.1038/nature11237 22859206 PMC4074413

[B43] Lundberg D. S. Yourstone S. Mieczkowski P. Jones C. D. Dangl J. L. (2013). Practical innovations for high-throughput amplicon sequencing. Nat. Methods 10, 999–1002. 10.1038/nmeth.2634 23995388

[B44] Maignien L. DeForce E. A. Chafee M. E. Eren M. A. Simmons S. L. (2014). Ecological succession and stochastic variation in the assembly of *Arabidopsis thaliana* phyllosphere communities. MBio 5, 1–10. 10.1128/mBio.00682-13 24449749 PMC3903271

[B45] Makhalanyane T. P. Valverde A. Gunnigle E. Frossard A. Ramond J. B. Cowan D. A. (2015). Microbial ecology of hot desert edaphic systems. FEMS Microbiol. Rev. 39, 203–221. 10.1093/femsre/fuu011 25725013

[B46] Manly B. (1986). Multivariate Statistical Methods: A Primer, 3rd Edn. London: Chapman and Hall, Ltd.

[B47] Marasco R. Rolli E. Ettoumi B. Vigani G. Mapelli F. Borin S.. (2012). A drought resistance-promoting microbiome is selected by root system under desert farming. PLoS ONE 7:e48479. 10.1371/journal.pone.0048479 23119032 PMC3485337

[B48] Massimo N. C. Nandi Devan M. M. Arendt K. R. Wilch M. H. Riddle J. M. Furr S. H.. (2015). Fungal endophytes in aboveground tissues of desert plants: infrequent in culture, but highly diverse and distinctive symbionts. Microb. Ecol. 70, 61–76. 10.1007/s00248-014-0563-6 25645243 PMC4457668

[B49] McGinnies W. (1979). Arid land ecosystems, in General Description of Desert Areas, Vol. I, eds Goodall D. W. Perry R. (Cambridge, UK: Cambridge University Press), 5–20.

[B50] Müller H. Berg C. Landa B. B. Auerbach A. Moissl-Eichinger C. Berg G. (2015). Plant genotype-specific archaeal and bacterial endophytes but similar *Bacillus* antagonists colonize Mediterranean olive trees. Front. Microbiol. 6:138. 10.3389/fmicb.2015.00138 25784898 PMC4347506

[B51] Nautiyal C. S. (1999). An efficient microbiological growth medium for screening phosphate solubilizing microorganisms. FEMS Microbiol. Lett. 170, 265–270. 10.1111/j.1574-6968.1999.tb13383.x 9919677

[B52] Nobel P. S. (2003). Environmental Biology of Agaves and Cacti. Cambridge: Cambridge University Press.

[B53] Nobel P. S. (2010). Desert Wisdom/Agaves and Cacti: CO2, Water, Climate Change. New York, NY; Bloomington, IN: iUniverse, Inc.

[B54] Noy-Meir I. (1973). Desert ecosystems: environment and producers. Annu. Rev. Ecol. Syst. 4, 25–51. 10.1146/annurev.es.04.110173.000325

[B55] Nunes da Rocha U. Cadillo-Quiroz H. Karaoz U. Rajeev L. Klitgord N. Dunn S.. (2015). Isolation of a significant fraction of non-phototroph diversity from a desert biological soil crust. Front. Microbiol. 6:227. 10.3389/fmicb.2015.00277 25926821 PMC4396413

[B56] Omoregie E. Crumbliss L. Bebout B. Zehr J. (2004). Determination of nitrogen-fixing phylotypes in *Lyngbya* sp and *Microcoleus chthonoplastes* cyanobacterial mats from Guerrero Negro, Baja California, Mexico. Appl. Environ. Microbiol. 70, 2119–2128. 10.1128/AEM.70.4.2119-2128.2004 15066804 PMC383108

[B57] Partida-Martínez L. P. Heil M. (2011). The Microbe-Free plant: fact or artifact? Front. Plant Sci. 2:100. 10.3389/fpls.2011.00100 22639622 PMC3355587

[B58] Peel M. C. Finlayson B. L. McMahon T. A. (2007). Updated world map of the Köppen-Geiger climate classification. Hydrol. Earth Syst. Sci. 11, 1633–1644. 10.5194/hess-11-1633-2007

[B59] Peiffer J. A. Spor A. Koren O. Jin Z. Tringe S. G. Dangl J. L.. (2013). Diversity and heritability of the maize rhizosphere microbiome under field conditions. Proc. Natl. Acad. Sci. U.S.A. 110, 6548–6553. 10.1073/pnas.1302837110 23576752 PMC3631645

[B60] Philippot L. Spor A. Hénault C. Bru D. Bizouard F. Jones C. M.. (2013). Loss in microbial diversity affects nitrogen cycling in soil. ISME J. 7, 1609–1619. 10.1038/ismej.2013.34 23466702 PMC3721106

[B61] Puente M. E. Bashan Y. Li C. Y. Lebsky V. K. (2004a). Microbial populations and activities in the rhizoplane of rock-weathering desert plants. I. Root colonization and weathering of igneous rocks. Plant Biol. 6, 629–642. 10.1055/s-2004-821100 15375735

[B62] Puente M. E. Li C. Y. Bashan Y. (2004b). Microbial populations and activities in the rhizoplane of rock-weathering desert plants. II. Growth promotion of cactus seedlings. Plant Biol. 6, 643–650. 10.1055/s-2004-821101 15375736

[B63] Puente M. E. Li C. Y. Bashan Y. (2009a). Endophytic bacteria in cacti seeds can improve the development of cactus seedlings. Environ. Exp. Bot. 66, 402–408. 10.1016/j.envexpbot.2009.04.007

[B64] Puente M. E. Li C. Y. Bashan Y. (2009b). Rock-degrading endophytic bacteria in cacti. Environ. Exp. Bot. 66, 389–401. 10.1016/j.envexpbot.2009.04.010

[B65] Quinn G. P. Keough M. J. (2002). Experimental Design and Data Analysis for Biologists, 1st Edn. New York, NY: Cambridge University Press.

[B66] R Core Team (2013). R: A Language and Environment for Statistical Computing. Vienna, Austria: R Foundation for Statistical Computing Available online at: http://www.r-project.org/

[B67] Rajeev L. Nunes da Rocha U. Klitgord N. Luning E. G. Fortney J. Axen S. D.. (2013). Dynamic cyanobacterial response to hydration and dehydration in a desert biological soil crust. ISME J. 7, 2178–2191. 10.1038/ismej.2013.83 23739051 PMC3806265

[B68] Rosenberg E. Sharon G. Atad I. Zilber-Rosenberg I. (2010). The evolution of animals and plants via symbiosis with microorganisms. Environ. Microbiol. Rep. 2, 500–506. 10.1111/j.1758-2229.2010.00177.x 23766221

[B69] Salazar J. R. Martínez-Vazquez M. Cespedes C. L. Ramírez-Apan T. Nieto-Camacho A. Rodríguez-Silverio J. (2011). Anti-inflammatory and cytotoxic activities of chichipegenin, peniocerol, and macdougallin isolated from *Myrtillocactus geometrizans* (Mart. ex Pfeiff.) Con. Z. Nat. 66 C, 24–30. 10.5560/ZNC.2011.66c0024 21476433

[B70] Santos-Zea L. Gutiérrez-Uribe J. A. Serna-Saldivar S. O. (2011). Comparative analyses of total phenols, antioxidant activity, and flavonol glycoside profile of cladode flours from different varieties of *Opuntia* spp. J. Agric. Food Chem. 59, 7054–7061. 10.1021/jf200944y 21598948

[B71] Sapkota R. Knorr K. Jørgensen L. N. O'Hanlon K. A. Nicolaisen M. (2015). Host genotype is an important determinant of the cereal phyllosphere mycobiome. New Phytol. 4, 1134–1144. 10.1111/nph.13418 25898906

[B72] Schlaeppi K. Dombrowski N. Oter R. G. Ver Loren van Themaat E. Schulze-Lefert P. (2014). Quantitative divergence of the bacterial root microbiota in *Arabidopsis thaliana* relatives. Proc. Natl. Acad. Sci. U.S.A. 111, 585–592. 10.1073/pnas.1321597111 24379374 PMC3896156

[B73] Singh B. Dawson L. A. Macdonald C. A. Buckland S. M. (2009). Impact of biotic and abiotic interaction on soil microbial communities and functions: a field study. Appl. Soil Ecol. 41, 239–248. 10.1016/j.apsoil.2008.10.003

[B74] Suryanarayanan T. S. Wittlinger S. K. Faeth S. H. (2005). Endophytic fungi associated with cacti in Arizona. Mycol. Res. 109, 635–639. 10.1017/S0953756205002753 16018319

[B75] Teather R. M. Wood P. J. (1982). Use of Congo red-polysaccharide interactions in enumeration and characterization of cellulolytic bacteria from the bovine rumen. Appl. Environ. Microbiol. 43, 777–780. 7081984 10.1128/aem.43.4.777-780.1982 PMC241917

[B76] Vandenkoornhuyse P. Quaiser A. Duhamel M. Le Van A. Dufresne A. (2015). The importance of the microbiome of the plant holobiont. New Phytol. 206, 1196–1206. 10.1111/nph.13312 25655016

[B77] Vazquez-Cruz A. M. Jimenez-Garcia S. N. Torres-Pacheco I. Guzman-Maldonado H. S. Guevara-Gonzalez R. G. Miranda-Lopez R. (2012). Effect of maturity stage and storage on flavor compounds and sensory description of berrycactus (*Myrtillocactus geometrizans*). J. Food Sci. 77, C366–C373. 10.1111/j.1750-3841.2012.02621.x 22429085

[B78] Vorholt J. A. (2012). Microbial life in the phyllosphere. Nat. Rev. Microbiol. 10, 828–840. 10.1038/nrmicro2910 23154261

[B79] Weinert N. Meincke R. Gottwald C. Heuer H. Schloter M. Berg G.. (2010). Bacterial diversity on the surface of potato tubers in soil and the influence of the plant genotype. FEMS Microbiol. Ecol. 74, 114–123. 10.1111/j.1574-6941.2010.00936.x 20698886

[B80] Wieland G. Neumann R. Backhaus H. (2001). Variation of microbial communities in soil, rhizosphere, and rhizoplane in response to crop species, soil type, and crop development. Appl. Environ. Microbiol. 67, 5849–5854. 10.1128/AEM.67.12.5849-5854.2001 11722945 PMC93382

[B81] Yang C. H. Crowley D. E. Borneman J. Keen N. T. (2001). Microbial phyllosphere populations are more complex than previously realized. Proc. Natl. Acad. Sci. U.S.A. 98, 3889–3894. 10.1073/pnas.051633898 11274410 PMC31148

[B82] Zarraonaindia I. Owens S. M. Weisenhorn P. West K. Hampton-marcell J. Lax S.. (2015). The soil microbiome influences grapevine-associated microbiota. MBio 6, 1–10. 10.1128/mBio.02527-14 25805735 PMC4453523

[B83] Zhang B. Li R. Xiao P. Su Y. Zhang Y. (2015). Cyanobacterial composition and spatial distribution based on pyrosequencing data in the Gurbantunggut Desert, Northwestern China. J. Basic Microbiol. 55, 1–13. 10.1002/jobm.201500226 26479723

