# Supplementary material for: The Cacti Microbiome: Interplay between Habitat-Filtering and Host-Specificity
Source: Front Microbiol. 2016 Feb 12;7:150. doi: 10.3389/fmicb.2016.00150 (PMC4751269; doi:10.3389/fmicb.2016.00150)
Supplement: Supplementary file 1 [file Presentation1.pdf]

Supplementary Material

The Cacti microbiome: interplay between habitat-filtering and host-specificity

Citlali Fonseca-García, Devin Coleman-Derr, Etzel Garrido, Axel Visel, Susannah G. Tringe and Laila P. Partida-Martínez*
*** Correspondence:** Laila P. Partida-Martinez (laila.partida@ira.cinvestav.mx)

1 Supplementary Data

Sampling and sequencing process

Sampling process in the field. Initially five plant compartments were considered for investigation: soil (bulk), rhizosphere (5 mm of soil adhering to the roots surface), root endosphere (the interior of the root), stem endosphere (the interior of the stem) and phyllosphere (stem surface), but the extreme dryness of the soils in which the Cacti specimens were collected meant in most cases, that very little soil adhered to the roots upon their removal from the ground. Therefore, besides the rhizosphere, we added a sample from soil close to the roots called root-zone soil, in case root exudates could have an impact on microbial communities even if not physically attached. Bulk soil samples were collected approximately 1 m away from each plant replicate by taking ~500 cm^3 soil from the top and up to 10-15 cm of depth and not displaying any visible influence of plants. The "root-zone soil" samples were collected by taking ~30-50 cm^3 soils up to 10 cm in proximity to the roots of the Cacti specimens. Both soil samples were stored in sterile Ziploc bags. Around ~30 g of roots and two stems (~20 cm each one) were collected using disinfected material (knifes, scissors, gloves, etc.) and placed in Ziploc bags (roots) or paper bags (stems) for transportation as shown in Figure S1. The apical stems and roots were collected from plants without signs of damage or disease (healthy). All collected samples were transported on ice to the laboratory, placed in a cold chamber (4°C) at arrival and processed the following day.

Roots were washed with 200 ml of 0.9% saline solution to collect the rhizosphere and the stems were washed with phosphate buffer (50mM KH_2PO_4, 50mM K_2HPO_4, 0.1% Triton X-100) to collect the phyllosphere from each biological replicate. Then, the wash buffers were independently concentrated to 50 ml and stored at -80 °C until DNA extraction. Washed roots and stems were sequentially surface disinfected in 70% ethanol for one minute, 5.5% NaOCl for 5 min and two washes of sterile water, all steps under sterile conditions. To prove the effectivity of the disinfection process, we plated 200 µl of the second wash water on Tryptone Soya Agar (TSA) and confirmed lack of microbial growth

after 7 days of incubation at room temperature (25°C). After surface disinfection, stem tissues were divided in three sections (basal, medium and apical) for their independent dissection. From each section, stem fragments of ~1 cm^3 were cut and placed in a 20% glycerol solution and stored at -80 °C. Root tissues were also dissected in small fragments of 1 cm length under sterile conditions and stored with 20% glycerol at -80 °C.

DNA extraction. DNA extractions were performed using different commercial kits and traditional protocols depending on the nature of each sample. DNA extractions from thawed bulk soil, root-zone soil and rhizosphere samples were done with the MoBioTM PowerSoil DNA Isolation Kit (MoBio Laboratories, Carlsbad, USA) because of its ability to remove humic acids and other compounds that inhibit the PCR reaction. For the phyllosphere, thawed samples were processed using the MoBioTM PowerWater DNA Isolation Kit. For the root endosphere samples, 100 mg of each frozen sample was grounded to a powder in liquid nitrogen with a sterile mortar and pestle and was then prepped following a CTAB genomic DNA extraction protocol by Edwards, (2001) with the following lysis solution (100mM Tris pH8, 20mM EDTA, 1.4 M NaCl, 2% Hexadecyltrimethylammonium bromide, 0.2% Beta mercaptoethanol). For the stem endosphere samples, 100 mg each of basal, medium and basal frozen samples were grounded to a powder in liquid nitrogen with a sterile mortar and pestle and DNA extraction was performed following the protocol by Lopes et al., (1995). Equimolar amounts of DNA obtained from the apical, medium and basal stem sections were pooled in a composite stem endosphere sample for the next steps. Finally, composite samples of each treatment (144 samples) were prepared by mixing equal amounts of DNA from each biological replicate to yield 48 pooled samples. DNA quantity and quality were assessed along the process with a Nanodrop ND-1000 (Thermo scientific, Wilmington, USA) spectrophotometer.

PCR amplification and sequencing process. The PCR amplification and sequencing processes were performed at the Joint Genome Institute. The microbial molecular markers employed in this study were the 16S rRNA V4 for bacterial/archaeal communities and the ITS2 for fungal communities. We employed the 515F (5’-GTGCCAGCMGCCGCGGTAA-3’) and 816R (5’-GGACTACHVGGGTWTCTAAT-3’) primer sets for the 16S rRNA V4 amplification. For ITS2 amplification, we used the ITS9F (5’-GAACGCAGCRAAIIGYGA-3’) and ITS4R (5’-TCCTCCGCTTATTGATATGC-3’) primer sets. In each PCR amplification using primers 515F-816R, we used PNA clamps to reduce chloroplast and mitochondrial contamination and increase the number of bacterial/archaeal reads from the root endosphere, stem endosphere and phyllosphere samples as described in Lundberg et al., (2013). The amplification reactions were performed in triplicate using ~10 ng of pool DNA template per reaction and were conducted in 96-well plate format. In order to determine the OTU measurability thresholds, we employed four negative controls and several technical replicates in each 96-well plate as

described in Coleman-Derr et al., (2016). The PCR conditions used were 94 °C for 3 min, followed by 30 cycles of 94 °C for 45s, 78 °C for 10s, 50 °C for 60s, and 72°C for 90s, and final extension to 72 °C for 10 min. Triplicate reactions from each sample were pooled and quantified with the Qubit High Sensitivity Assay kit (Life Technologies) on a Turner Biosystems fluorescence plate reader (Promega, Madison, WI, USA). Sets of 96 barcoded PCR products pooled in equimolar ratios and cleaned up using the AMPureXP magnetic beads (Beckman-Coulter, Indianapolis, IN, USA). Paired-end 2 x 250bp sequencing of the barcoded amplicons was performed on a MiSeq machine running v2 chemistry (Illumina Inc, San Diego CA, USA). Samples from Cacti were processed together with the samples derived from *Agave* species reported by Coleman-Derr et al., (2016) .

Data processing. The raw Fastq reads generated from Cacti samples were processed using a custom pipeline developed at the Joint Genome Institute. First, Illumina adapter sequences and PhiX sequencing control was removed from raw reads. Primer sequences were trimmed from the 5' end and low-quality bases were trimmed from the 3' end of reads prior to assembly of read1 and read2 with either FLASH (Magoč and Salzberg, 2011) for 16S data or Pandaseq (Masella et al., 2012) for ITS2 data. After that, 1,311,071 bacterial/archaeal and 2,752,860 fungal high-quality merged reads were obtained (Figure S3). These reads were clustered using the UPARSE pipeline (Edgar, 2013) to yield 40,759 bacterial/archaeal and 25,871 fungal OTUs at 97 and 95% identity, respectively. While 97% cut-off is generally sufficient for properly identifying fungal species using either ITS1 or ITS2, some species are split into two or more groups at this level of clustering (Blaalid et al., 2013). The JGI uses 95% for all its fungal ITS2 analyses, which will keep more fungal species from being split into two or more units, but at the expense of some species clustering together with other species.

Taxonomic lineage were assigned to each OTU using the RDP Naïve Bayesian Classifier (Wang et al., 2007) with custom reference databases. For the 16S V4 data, this database was compiled from the May 2013 version of the GreenGenes 16S database (DeSantis et al., 2006), the Silva 16S database (Quast et al., 2013) and additional manually-curated 16S sequences, trimmed to the V4 region. For the ITS2 data, this database was built from the UNITE database (Koljalg, 2013). After that the taxonomies were assigned to each OTU, we discarded some OTUs considering the following criteria: (1) OTUs that were not assigned a Kingdom level RDP classification score of at least 0.5, (2) OTUs that were not assigned to Kingdom Bacteria or Archaea for the 16S datasets, and (3) all OTUs that were not assigned to Kingdom Fungi for ITS2 datasets. Using several technical replicates included on each sequenced 96-well plate, we calculated a threshold for determining technical reproducibility for these OTUs using the progressive drop-out analysis described in Lundberg et al., (2013) (Coleman-Derr et al., 2016 Figure S2). This threshold determines the minimum cumulative read count across all plates for an OTU to be included in the

analysis, and was determined to be at least 7 reads in at least 5 samples for the 16S dataset and at least 2 reads in at least 5 samples for the ITS2 dataset. These criteria yielded 4,012 and 3,541 measurable OTUs for bacteria/archaea and fungi, respectively for most downstream analyses (these OTUs are considered as the measurable OTUs and were deposited under Accession Numbers KU536055 - KU539595). Only for diversity analyses and in order to account for differences in sequencing read depth across samples, samples were randomly subsampled (rarefied) to 275 and 375 reads per sample in the bacterial/archaeal and fungal datasets, respectively. OTU measurable tables and metadata can be found as Supplementary Material (Data Sheet 1). All Illumina sequences related to this project were deposited under SRA accession: SRP068631.

In vitro biochemical characterization of seed-borne bacteria associated with *M. geometrizans* and *O. robusta*

In vitro tolerance to water stress

Growth capacity under water reduction. Bacterial cells were grown overnight or up to one day in Tryptone Soya Broth (TSB) at 28 °C and 150 rpm. Optical density of cultures was then measured at 600 nm (OD_{600nm}) and adjusted to 1.0 in all cases. Subsequently, 10 µl of serial dilutions of 10^2, 10^4, 10^6 and 10^8 of adjusted cultures were poured in 10% TSA plates containing 405 g/l, 202.5 g/l, 101.25 g/l and 0 g/l (control) of sorbitol. Finally, the plates were incubated at 28 °C for a period of five days and the growth was monitored every 24 hrs (Kavamura et al., 2013).

Exopolysaccharides production. 20 µl of grown and adjusted cultures (OD_{600nm}= 1.0) were adsorbed on filter paper discs placed in the selective media (yeast extract 2%, 1.5% K_2HPO_4, 0.02% $MgSO_4$, 0.0015% $MnSO_4$, 0.0015% $FeSO_4$, 0.003% $CaCl_2$, 0.0015% NaCl, 1.5% agar and 10% glucose) previously adjusted to pH 6.5 and 7.5. As control, growth on TSA media was monitored. Exopolysaccharide production is characterized by the presence of a halo with silty appearance. Thus, the halo size and its appearance were monitored every 24 hrs for a period of 5 days (Paulo et al., 2012).

Exopolysaccharide staining with alcian blue. A loop of cells growing on the exopolysaccharides selective media was taken and suspended in 1 ml of a solution of Alcian Blue (alcian blue and acetic acid, 1:8 (v/v)). The suspension was mixed and incubated one hour at room temperature. Centrifugation followed at 12 krpm for 5 min; the supernatant discarded and the pellet washed with 200 µl of sterile distilled water. A second round of centrifugation took place at 10 krpm for 5 min; the supernatant removed and the pellet resuspended in 20 µl of sterile distilled water. 10 µl of the staining was observed under a light microscope at a magnification of 100X (Leica DM 750, Leica Microsystems).

The alcian blue dye binds to the carboxyl groups of the polysaccharides without binding to bacterial cell walls, allowing a clear differentiation (Paulo et al., 2012).

Plant growth promotion traits by direct mechanisms

Nitrogen fixation capacity in solid and semi-solid media. 10 µl of serial dilutions of 10^2, 10^4, 10^6 and 10^8 of grown and adjusted cultures (OD_{600nm}= 1.0) were inoculated in Winogradsky media at pH 7 (100 ml of Winodradsky salts (5 g of K_2HPO_4, 2.5 g of $MgSO_4 \cdot 7H_2O$, 2.5 g of NaCl, 0.05 g of $Fe(SO_4)_3$ and 0.05 g of $MnS0_4$ for gauging one liter), 0.5 g of CaCO3, 0.5% of bromothymol blue (as an indicator of pH), 10 g of carbon source and 8 g of agar to one liter of medium) with different carbon sources (glucose, sucrose and sucrose/mannitol). We used growth on TSA as control for each strain. Nitrogen fixation is characterized by the formation of halo with a shift from blue-green coloration to yellow, this due to acidification of the culture medium. The size and coloration was measured and recorded every 24 hrs for a period of 5 days (Hardy et al., 1968). Semi-solid media is exactly as above reported, but agar concentration was lowered to 2 g per liter instead of 8. Evaluation in semi-solid media allowed the determination of the oxygen requirements of evaluated strains. As in solid medium, nitrogen fixation is characterized by the formation of a color shift from blue-green to yellow. Coloration and position of bacterial growth (superficial, middle or bottom of the tube) were monitored every 24 hrs for a period of three days (Hardy et al., 1968).

Indole acetic acid (IAA) production. 100 µl of grown and adjusted bacterial cultures (OD_{600nm}= 1.0) were inoculated into 10 ml of 10% TSB media supplemented with 5 mM L-tryptophan and incubated in dark at 28 °C for a period of 48 hrs. Then, the cultures were centrifuged at 10 krpm for 5 min and 750 µl of the supernatant were treated with 750 µl of Salkowski reagent (50 ml of perchloric acid (35%) and 1 ml of $FeCl_3$ (0.5 M)) for 30 min under protection from light. As negative control, 750 µl of culture without bacterial inoculation was used. Finally, the optical density was measured in a spectrophotometer at 530 nm (Beckman DU640). The production of a pink to red color indicates the production of IAA (Gordon and Weber, 1951). We developed a calibration curve with commercial IAA using concentrations of 3-50 ng/ml.

Phosphate solubilizing capacity. We employed the colorimetric method Nautiyal (1999) with some modifications in order to detect available phosphate. 100 µl of grown and adjusted bacterial cultures (OD_{600nm}= 1.0) were incubated in 10 ml of NBRIP (National Botanical Research Institute's Phosphate) media (1% glucose, 0.5% $Ca_3(PO_4)_2$, 0.5% $MgCl_2$, 0.02% KCl, 0.025% $MgSO_4 \cdot 7H_2O$ and 0.01% $(NH_4)_2SO_4$) at 28 °C, 180 rpm for 15 days. Afterwards, 1 ml of each sample was taken, transferred to 1.5 ml tubes and centrifuged at 10 krpm for 5 min. Then, 145 µl of supernatant was collected and addition of 570 µl of sterile distillated water and 285 µl of molybdate-vanadate (5% ammonium

molybdate, 0.25% ammonium vanadate, 1:1 (v/v)) followed. The mixture was incubated for 10 min at room temperature. As negative control, we used uninoculated NBRIP media. Finally, the optical density was measured in a spectrophotometer at 420 nm (Beckman DU640). We developed a calibration curve with KH_2PO_4 using concentrations of 50-350 ng/ml.

Siderophores detection. 10 µl of grown and adjusted bacterial cultures (OD_{600nm}= 1.0) were inoculated into CAS media and incubated at 28 °C until growth could be observed. The CAS medium uses a ferrichrome complex which changes color as a result of iron loss. Siderophores that have more affinity for iron chromogen can capture the ferry iron-chromogen complex, turning dye color from blue to yellow-orange (De los Santos-Villalobos et al., 2012).

Plant growth promotion traits by indirect mechanisms

Ammonia production. 100 µl of grown and adjusted bacterial cultures (OD_{600nm}= 1.0) were inoculated in 10 ml peptone media and incubated for 48 hrs at 28 °C. Then, from each culture 1 ml was taken and transferred to a 1.5 ml microcentrifuge tube and 50 µl of reagent Nessler (10% HgI_2, 7% KI and 50% NaOH (32%)) were added. Mixtures developing a light yellow color indicate a small amount of ammonia production, whereas a strong yellow to brown color indicate a high production of ammonia together with a precipitate resulting from the reaction of ammonia with mercuric iodide (Cappuccino and Sherman, 1992).

Production of hydrogen cyanide (HCN). The strains were streaked on TSA media supplemented with 10% glycine (4.4 g/l) and incubated at 28 °C. After 24 hrs of incubation and under sterile conditions, a sterile plate-sized filter paper soaked with a solution of 0.5% picric acid and 2% Na_2CO_3 was placed in each petri dish. The plates were sealed and incubated with the filter paper up to 48 hrs. Hydrogen cyanide production is considered positive if paper filter turns orange to red-brown. Color and cell growth were monitored every 24 hrs (Bakker and Schippers, 1987).

Capacity cellulose hydrolysis. 20 µl of grown and adjusted bacterial cultures (OD_{600nm}= 1.0) were adsorbed on filter paper discs placed in Mandels media (2.0 g of KH_2PO_4, 0.4 g of $CaCl_2$, 5.0 mg of $FeSO_4 \cdot 7H_2O$, 1.4 g of $(NH_4)_2SO_4$, 0.3 g urea, 0.3 g of $MgSO_4 \cdot 7H_2O$, 1.6 mg of $MnSO_4 \cdot H_2O$, 1.4 g of $ZnSO_4 \cdot 7H_2O$, 20 mg of $CoCl_2$, 1.0 g Tween 80, 25.0 g of peptone, 1.0 g of carboxymethylcellulose and 12g of agar). Then, the plates were incubated at 28 °C until radial growth could be observed (Teather and Wood, 1982). Congo red staining was performed at 1% (w/v) trying to cover the entire plate. After 15 min, excess dye was removed and 1M NaCl was added for 15 min. Finally, the presence of halos of hydrolysis was analyzed and recorded.

References

Bakker, A. W., and Schippers, B. (1987). Microbial cyanide production in the rhizosphere in relation to potato yield reduction and *Pseudomonas* SPP-mediated plant growth-stimulation. *Soil Biol. Biochem.* 19, 451–457. doi:10.1016/0038-0717(87)90037-X.

Blaalid, R., Kumar, S., Nilsson, R. H., Abarenkov, K., Kirk, P. M., and Kauserud, H. (2013). ITS1 versus ITS2 as DNA metabarcodes for fungi. *Mol. Ecol. Resour.* 13, 218–224. doi:10.1111/1755-0998.12065.

Cappuccino, J. C., and Sherman, N. (1992). *Microbiology: A laboratory manual*. Third. New York: Benjamin/Cummings Publishing Company.

Coleman-Derr, D., Desgarennes, D., Fonseca-Garcia, C., Gross, S., Clingenpeel, S., Woyke, T., et al. (2016). Plant compartment and biogeography affect microbiome composition in cultivated and native Agave species. *New Phytol.* 209, 798. doi:doi:10.1111/nph.13697.

DeSantis, T. Z., Hugenholtz, P., Larsen, N., Rojas, M., Brodie, E. L., Keller, K., et al. (2006). Greengenes, a chimera-checked 16S rRNA gene database and workbench compatible with ARB. *Appl. Environ. Microbiol.* 72, 5069–5072. doi:10.1128/AEM.03006-05.

Edgar, R. C. (2013). UPARSE: highly accurate OTU sequences from microbial amplicon reads. *Nat. Methods* 10, 996–8. doi:10.1038/nmeth.2604.

Edwards, K. J. (2001). "Miniprep procedures for the isolation of plant DNA," in *Molecular Tools for Screening Biodiversity*, eds. A. Karp, D. S. Ingram, and P. G. Isaac (The Netherlands: Kluwer Academic Publishers), 22–23.

Gordon, A. S., and Weber, R. P. (1951). Colorimetric estimation of indoleacetic acid. *Plant Physiol.* 26, 192–195. doi:10.1104/pp.26.1.192.

Hardy, R. W., Holsten, R. D., Jackson, E. K., and Burns, R. C. (1968). The acetylene-ethylene assay for N2 fixation: laboratory and field evaluation. *Plant Physiol.* 43, 1185–1207.

Kavamura, V. N., Santos, S. N., Da Silva, J. L., Parma, M. M., Ávila, L. A., Visconti, A., et al. (2013). Screening of Brazilian cacti rhizobacteria for plant growth promotion under drought. *Microbiol. Res.* 168, 183–191. doi:10.1016/j.micres.2012.12.002.

Koljalg, U. (2013). Towards a unified paradigm for sequence-based identification of fungi. *Mol. Ecol.* 22, 5271–5277. doi: 10.1111/mec.12481.

Lopes, M. A., Takasaki, K., Bostwick, D. E., Helentjaris, T., and Larkins, B. A. (1995). Identification of two opaque modifier loci Quality Protein Maize. *Mol. Gen. Genet.*

247, 603–613.

De los Santos-Villalobos, S., Barrera-Galicia, G. C., Miranda-Salcedo, M. A., and Peña-Cabriales, J. J. (2012). *Burkholderia cepacia* XXVI siderophore with biocontrol capacity against *Colletotrichum gloeosporioides*. *World J. Microbiol. Biotechnol.* 28, 2615–2623. doi:10.1007/s11274-012-1071-9.

Lundberg, D. S., Yourstone, S., Mieczkowski, P., Jones, C. D., and Dangl, J. L. (2013). Practical innovations for high-throughput amplicon sequencing. *Nat. Methods* 10, 999–1002. doi:10.1038/nmeth.2634.

Magoč, T., and Salzberg, S. L. (2011). FLASH: fast length adjustment of short reads to improve genome assemblies. *Bioinformatics* 27, 2957–2963. doi:10.1093/bioinformatics/btr507.

Masella, A. P., Bartram, A. K., Truszkowski, J. M., Brown, D. G., and Neufeld, J. D. (2012). PANDAseq: paired-end assembler for illumina sequences. *BMC Bioinformatics* 13, 31. doi:10.1186/1471-2105-13-31.

Paulo, E. M., Boffo, E. F., Branco, A., Valente, A. M. M. P., Melo, I. S., Ferreira, A. G., et al. (2012). Production, extraction and characterization of exopolysaccharides produced by the native *Leuconostoc pseudomesenteroides* R2 Strain. *Ann. Brazilian Acad. Sci.* 84, 495–507. doi:10.1590/S0001-37652012000200018.

Quast, C., Pruesse, E., Yilmaz, P., Gerken, J., Schweer, T., Yarza, P., et al. (2013). The SILVA ribosomal RNA gene database project: improved data processing and web-based tools. *Nucleic Acids Res.* 41, D590–D596. doi:10.1093/nar/gks1219.

Teather, R. M., and Wood, P. J. (1982). Use of Congo red-polysaccharide interactions in enumeration and characterization of cellulolytic bacteria from the bovine rumen. *Appl. Environ. Microbiol.* 43, 777–780.

Wang, Q., Garrity, G. M., Tiedje, J. M., and Cole, J. R. (2007). Naïve Bayesian classifier for rapid assignment of rRNA sequences into the new bacterial taxonomy. *Appl. Environ. Microbiol.* 73, 5261–5267. doi:10.1128/AEM.00062-07.

1. Supplementary Figures and Tables

2.1 Suplementary Figures

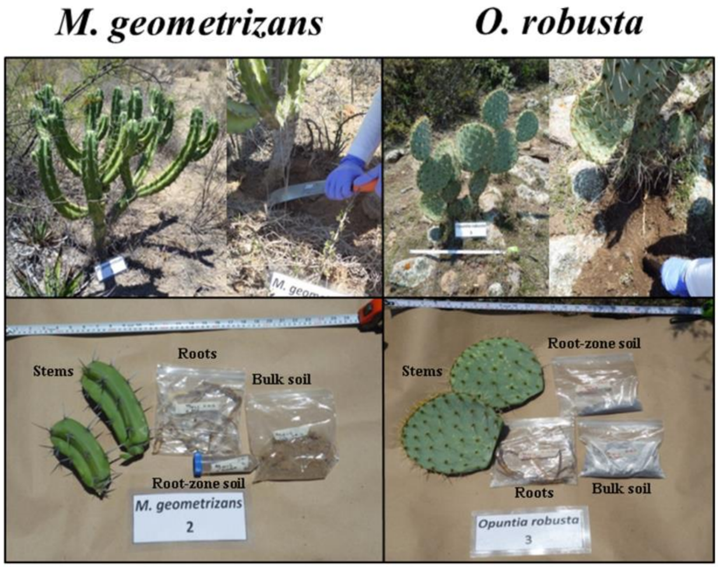

Figure S1. Examples of stems, roots and soils recovered from *M. geometrizans* and *O. robusta* in the field.

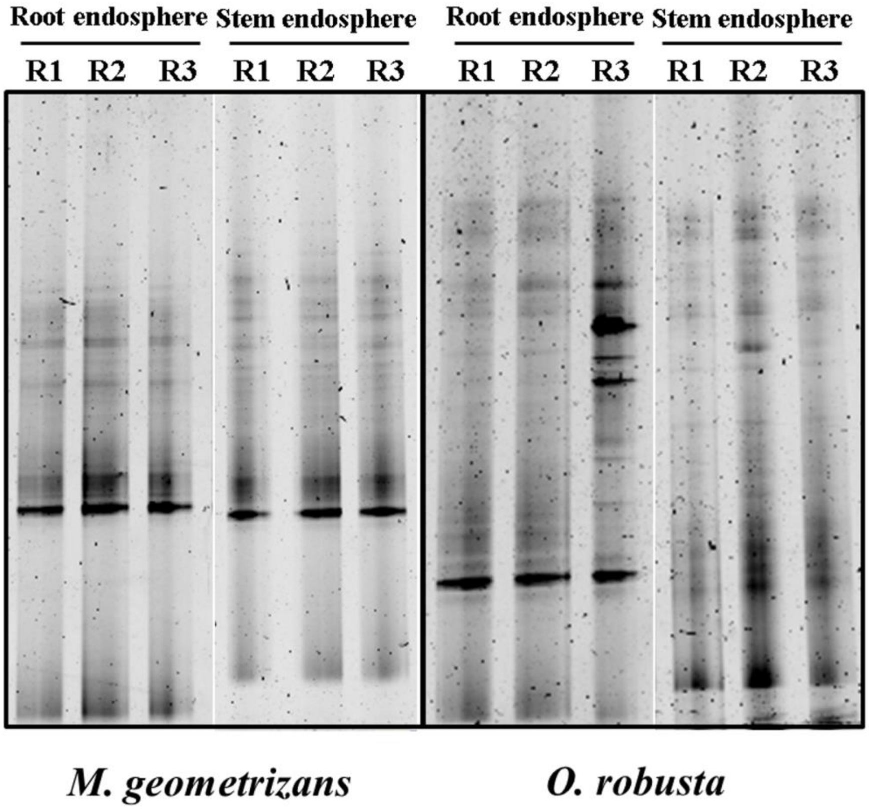

Figure S2. DGGE profiles of 16S rRNA gene fragments (Region V6-V8) from the endophytic bacterial communities associated with *M. geometrizans* and *O. robusta* in 2012. R: biological replicate.

(A)

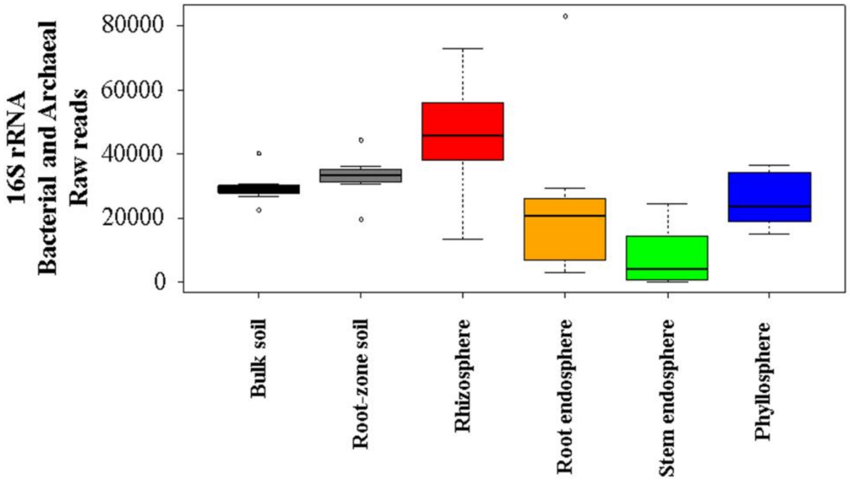

(B)

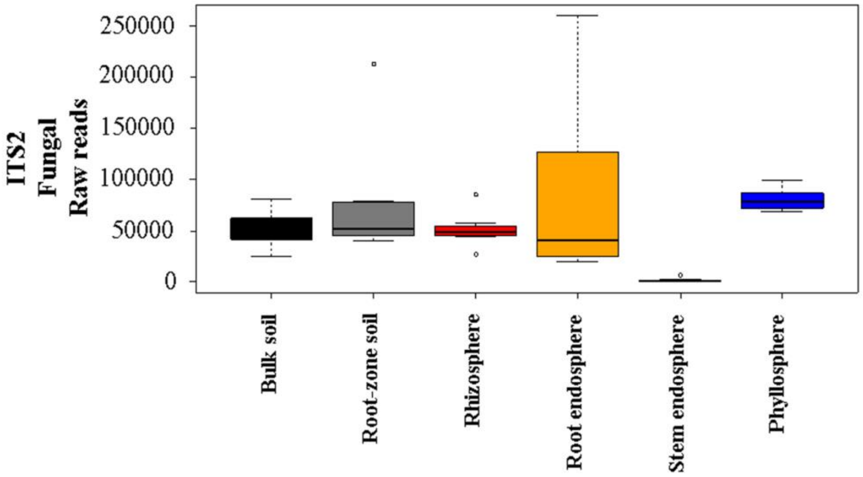

Figure S3. Raw read counts among the 48 pooled Cacti samples grouped by plant compartment. Boxplots of raw read counts per plant compartment for (**A**) bacterial/archaeal 16S rRNA gene and (**B**) fungal ITS2 datasets. Readcounts represented here are taken after removal of non-target sequences (chloroplast, mitochondrial, etc.), but prior to applying technical measurability thresholds. The center of each box represents the median value, while the upper and lower limit of the box represent the first and third quartile. The whiskers represent +/- 1.5 times the interquartile range (IQR), and outliers beyond this range are indicated as open circles.

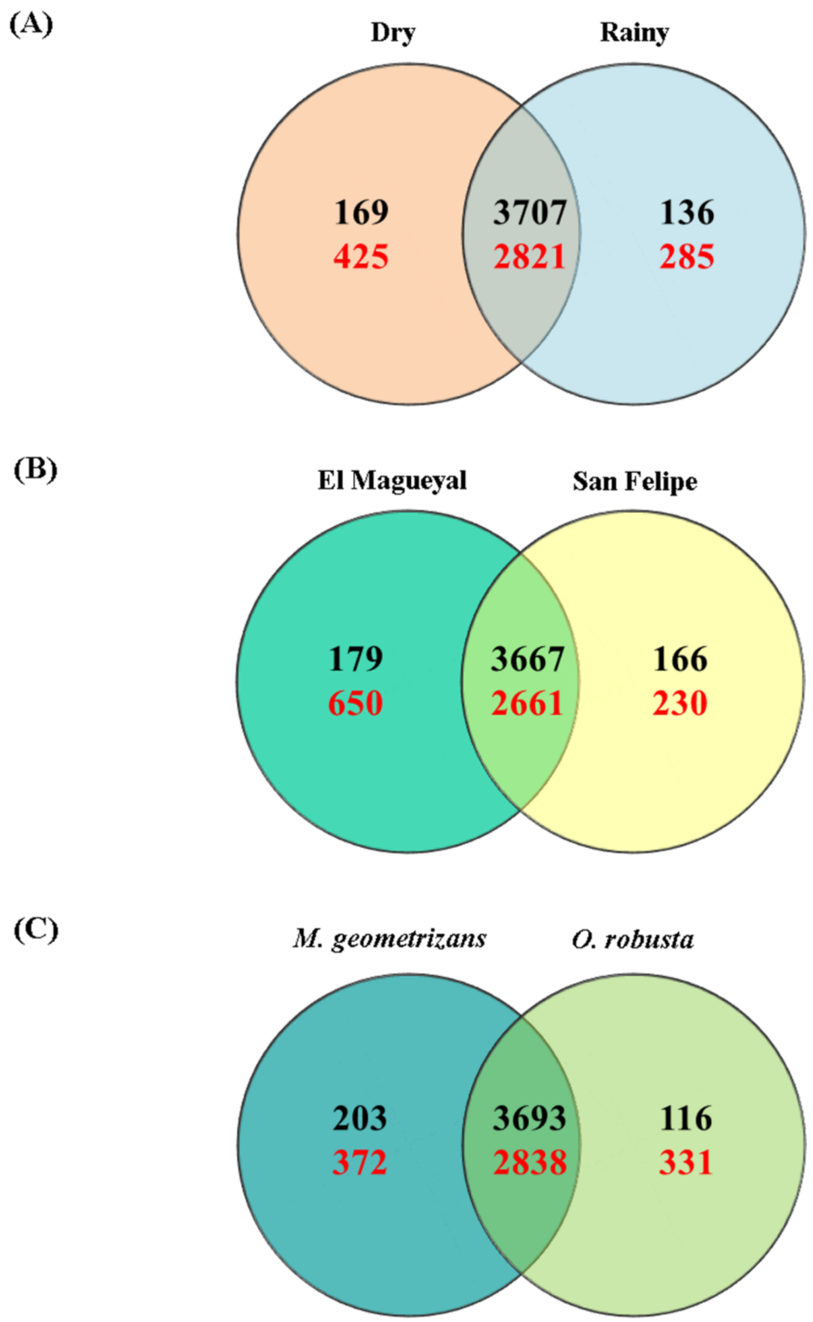

Figure S4. Venn diagrams showing the distribution of measurable OTUs by factor analyzed considering all 48 pooled samples. The diagrams indicate the number of bacterial/archaeal (top, in black) and fungal (botton, in red) OTUs between (**A**) dry and rainy season, (**B**) El Magueyal and San Felipe, and (**C**) *M. geometrizans* and *O. robusta*.

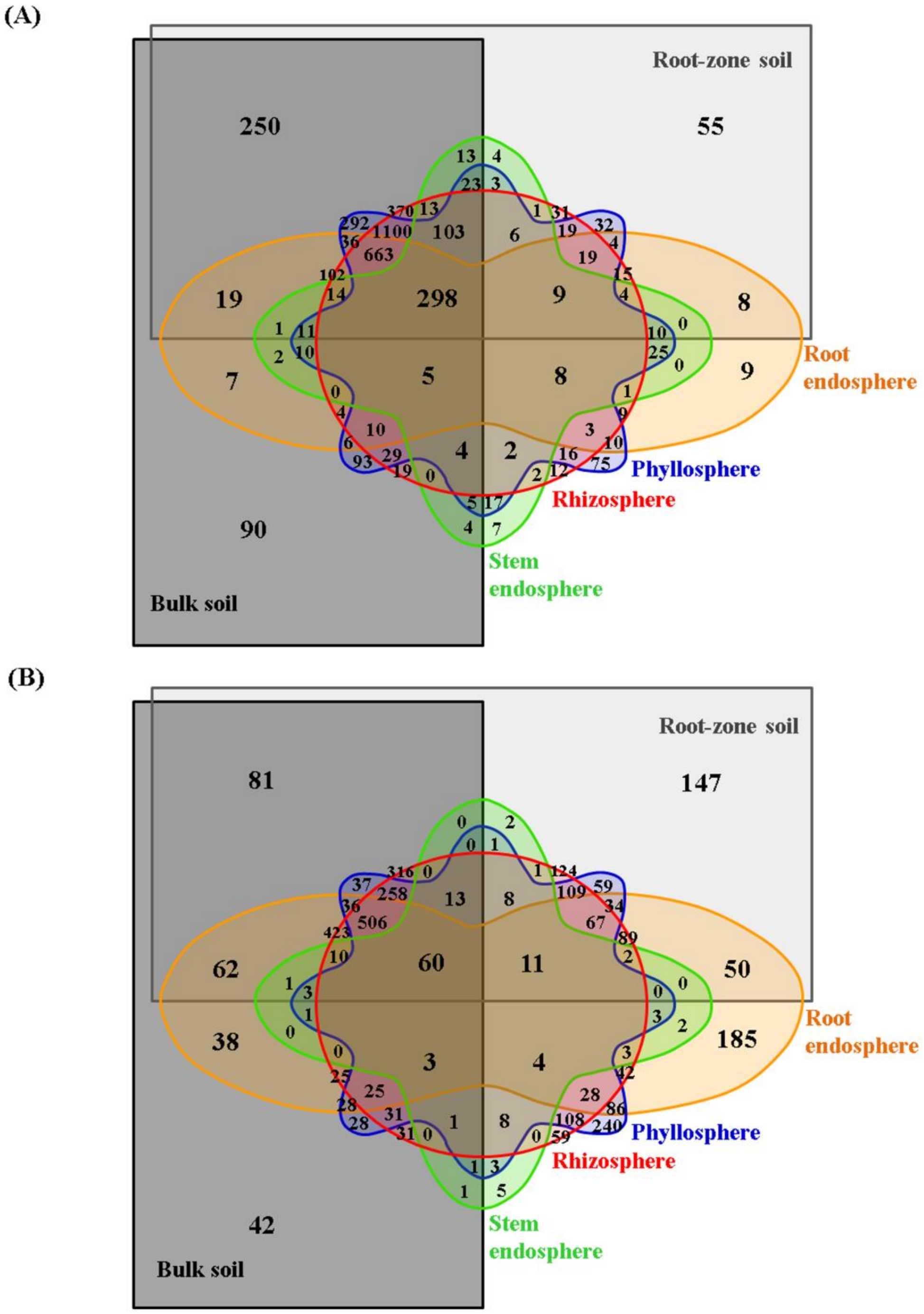

Figure S5. Venn diagrams showing the distribution of measurable (**A**) bacterial/archaeal and (**B**) fungal OTUs by plant compartment.

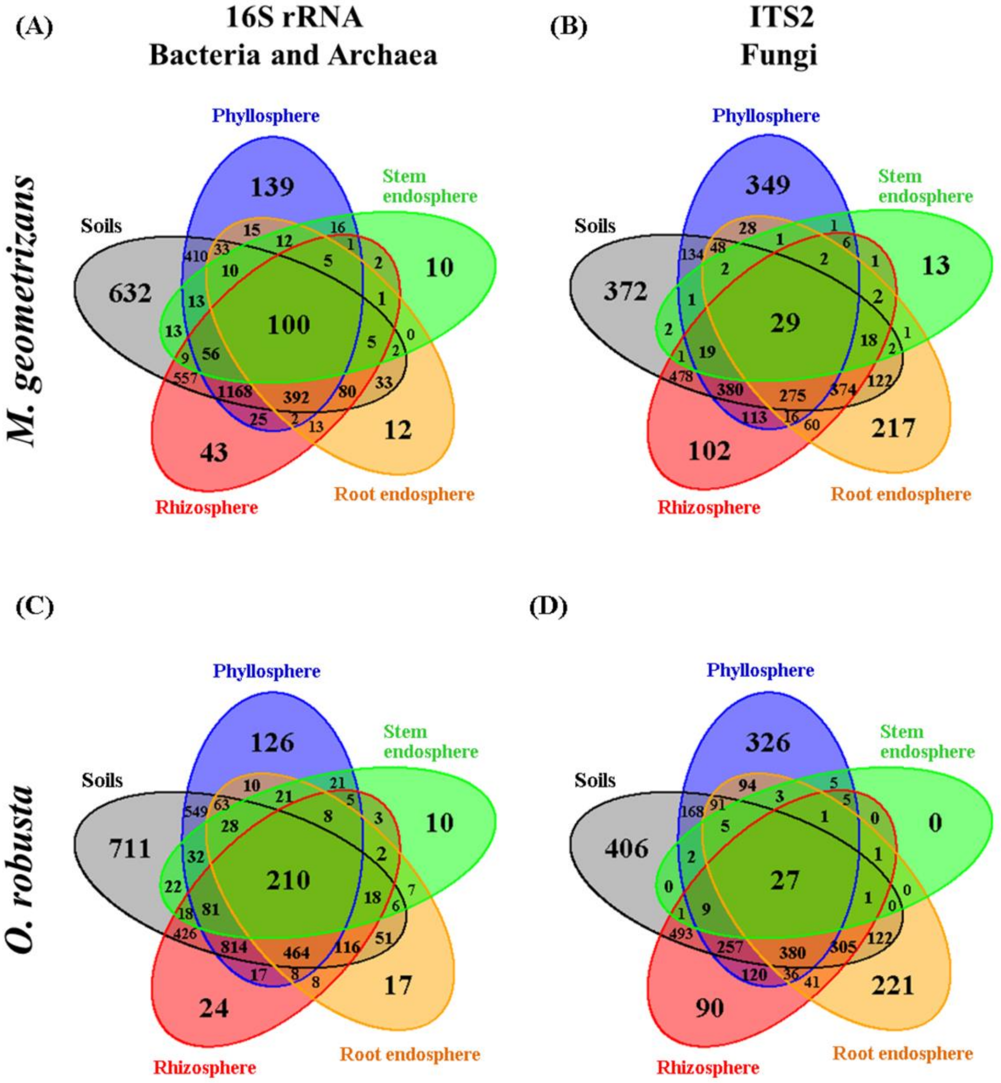

Figure S6. Venn diagrams showing the distribution of (**A, C**) bacterial/archaeal and (**B, D**) fungal measurable OTUs associated with (**A, B**) *M. geometrizans* and (**C, D**) *O. robusta* by plant compartment. The root-zone and bulk soils were grouped together as they showed little differences among them.

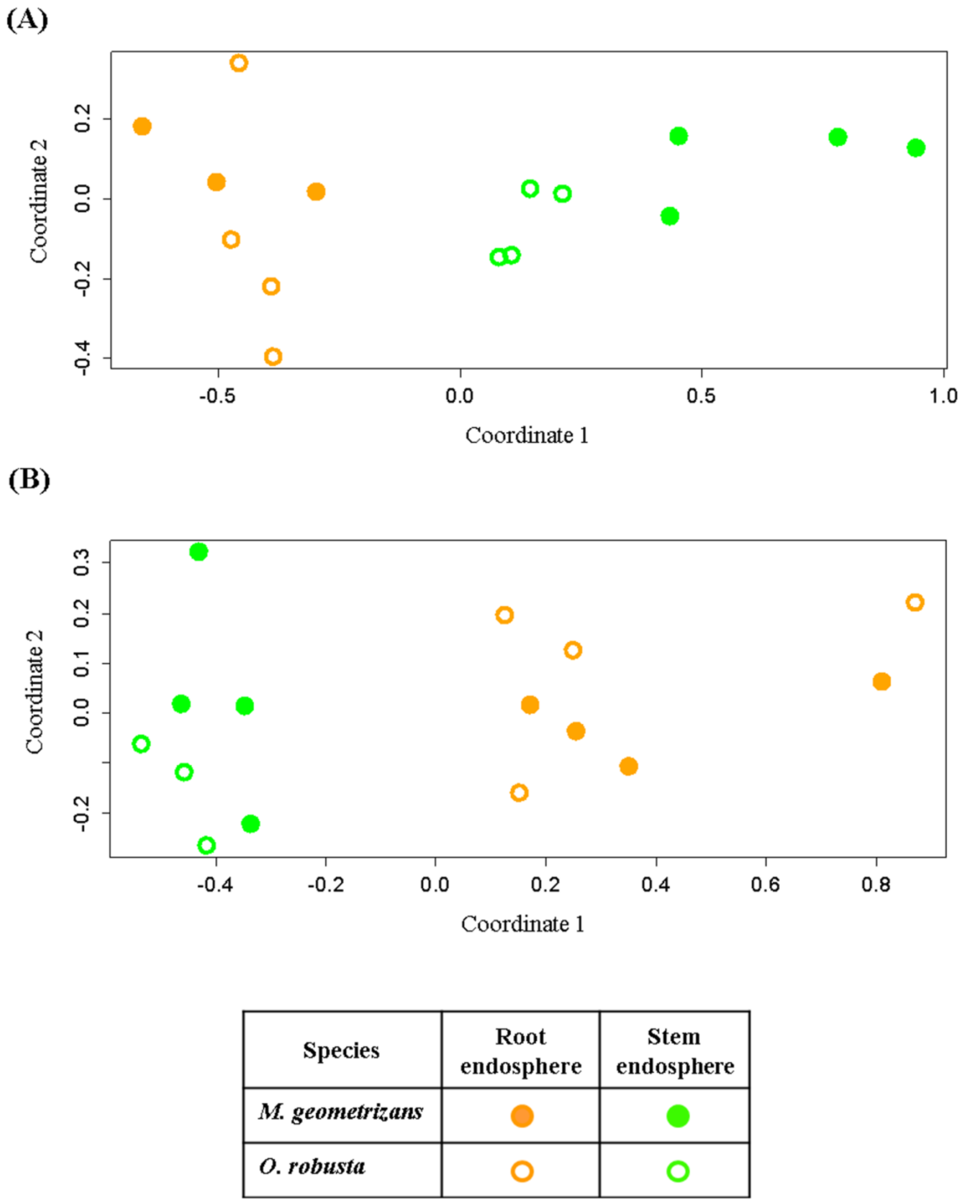

Figure S7. Nonmetric multidimensional scaling (NMDS) plots for Bray-Curtis distances of the (**A**) bacterial/archaeal (S = 0.07) and (**B**) fungal (S = 0.09) endophytic communities associated with *M. geometrizans* (shaded symbols) and *O. robusta* (empty symbols).

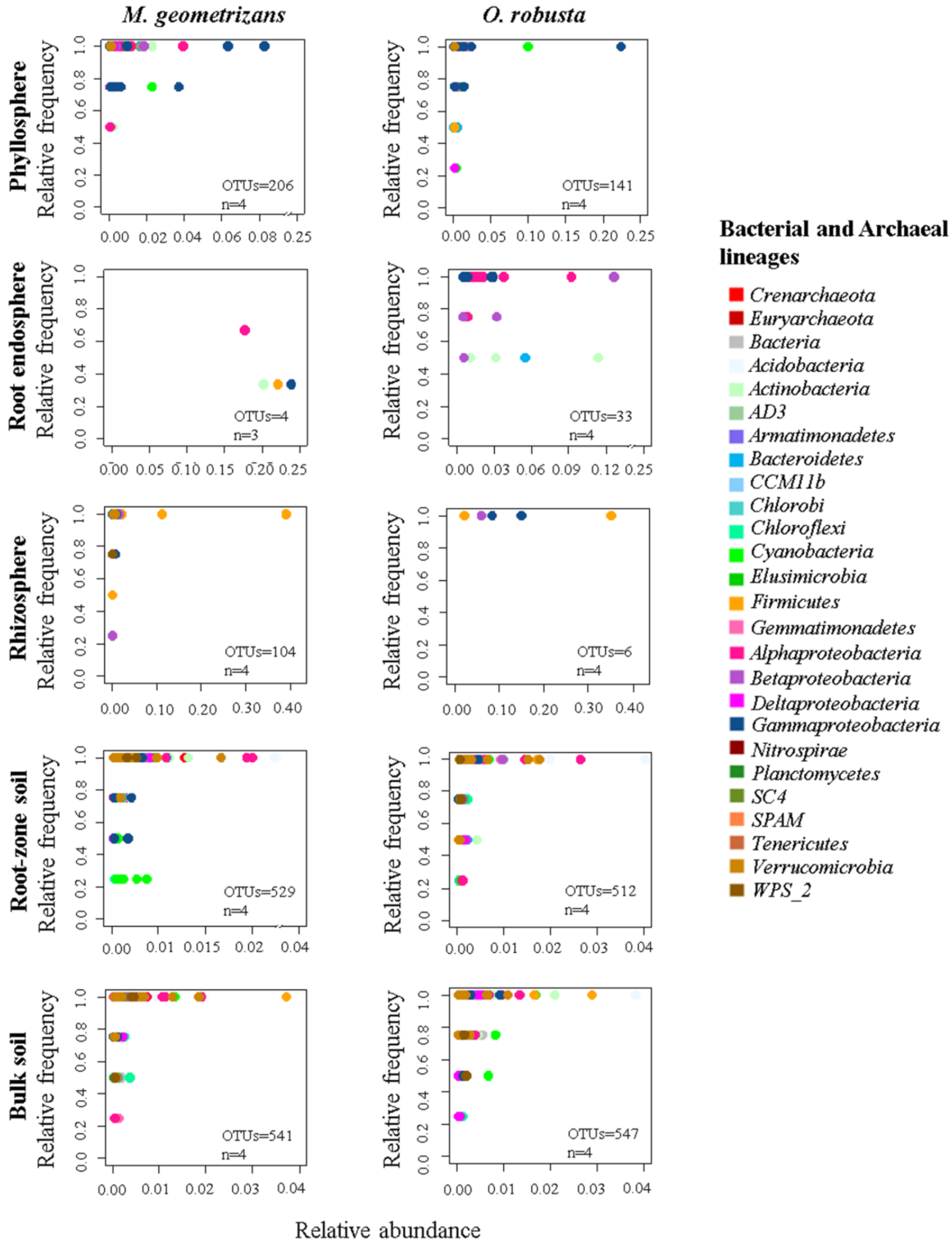

Figure S8. Pareto analysis of the bacterial/archaeal communities observed in *M. geometrizans* (left) and *O. robusta* (right), where 20% or less of the total number of OTUs account for 80% of the accumulated relative abundance. The number of OTUs and the number of samples (n) are indicated in the bottom right corner of each plot.

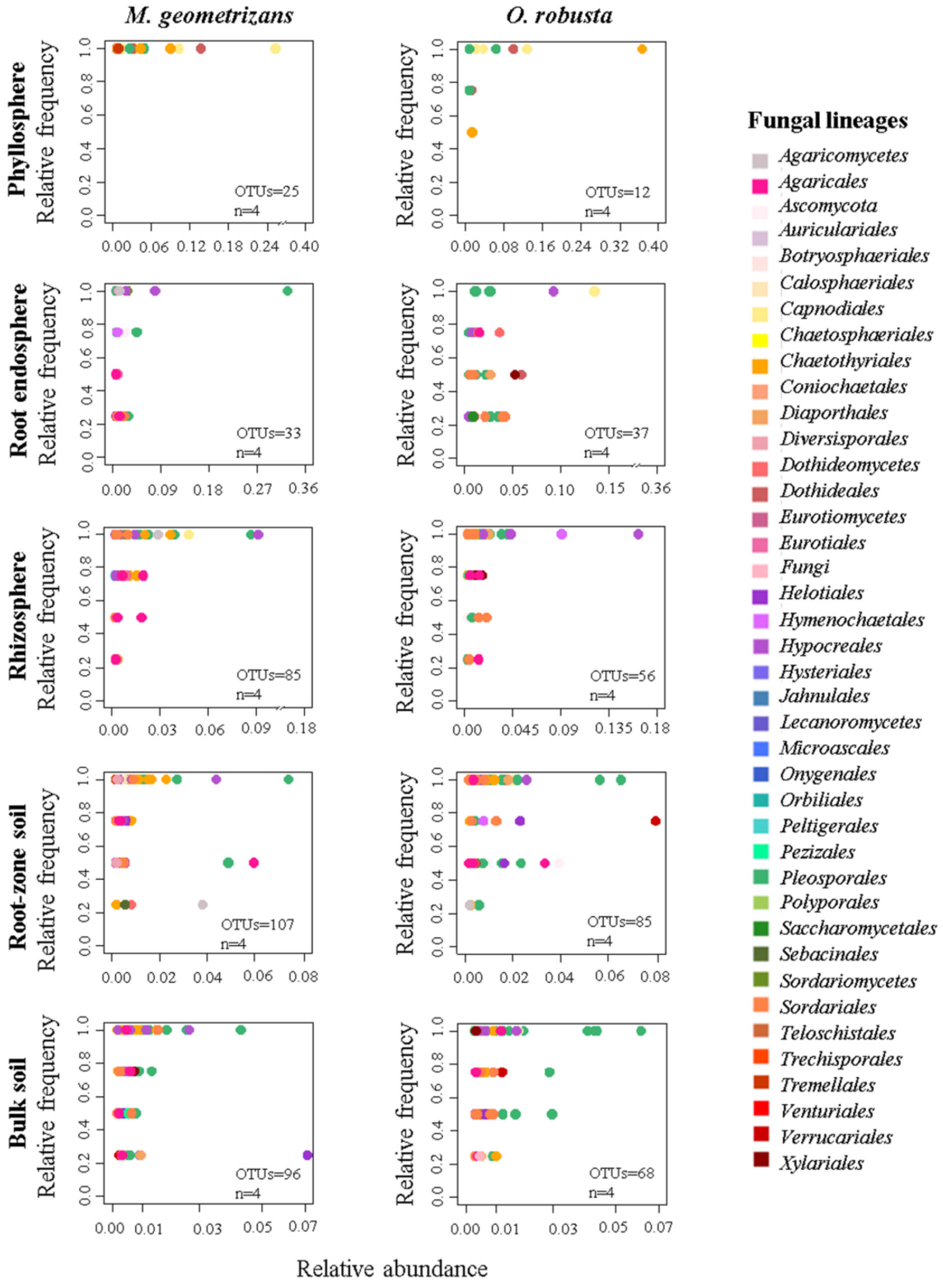

Figure S9. Pareto analysis of the fungal communities observed in *M. geometrizans* (left) and *O. robusta* (right), where 20% or less of the total number of OTUs account for 80% of the accumulated relative abundance. The number of OTUs and the number of samples (n) are indicated in the bottom right corner of each plot.

2.2 Supplementary Tables

Table S1. Plant characteristics and geographic location.

ID	Height (cm)	Diameter (cm)	H/D	Neighboring plants	Collection date	Season	Site	North	West	Altitude (masl)
Mg $_{M\ 1\text{-}1}$	71	32	2.22	Mg, As	04.06.12	Dry	Ma	21°05.403	100°17.559	2245
Mg $_{M\ 1\text{-}2}$	90	65	1.38	Oi	04.06.12	Dry	Ma	21°05.219	100°17.737	2185
Mg $_{M\ 1\text{-}3}$	74	69	1.07	Mg, As	04.06.12	Dry	Ma	21°04.097	100°16.100	2165
Mg $_{Sf\ 1\text{-}1}$	82	79	1.04		30.05.12	Dry	SF	21°38.528	101°03.409	1894
Mg $_{Sf\ 1\text{-}2}$	102	121	0.84	As, *Prosopis* sp.	30.05.12	Dry	SF	21°39.111	101°01.325	1887
Mg $_{Sf\ 1\text{-}3}$	70	60	1.17	*Prosopis* sp.	30.05.12	Dry	SF	21°40.212	101°02.719	1881
Mg $_{M\ 2\text{-}1}$	89	82	1.08		10.10.12	Rainy	Ma	21°05.194	100°17.716	2174
Mg $_{M\ 2\text{-}2}$	92	89	1.03	As, *Opuntia* sp.	10.10.12	Rainy	Ma	21°04.111	100°16.115	2173
Mg $_{M\ 2\text{-}3}$	73	59	1.24		10.10.12	Rainy	Ma	21°04.113	100°16.111	2172
Mg $_{Sf\ 2\text{-}1}$	103	79	1.30		12.10.12	Rainy	SF	21°40.280	101°02.667	1875
Mg $_{Sf\ 2\text{-}2}$	67	62	1.08		12.10.12	Rainy	SF	21°40.266	101°02.729	1879
Mg $_{Sf\ 2\text{-}3}$	77	98	0.79		12.10.12	Rainy	SF	21°38.568	101°03.275	1885
Or $_{M\ 1\text{-}1}$	63	72	0.88		04.06.12	Dry	Ma	21°05.398	100°17.541	2241
Or $_{M\ 1\text{-}2}$	46	65	0.71		04.06.12	Dry	Ma	21°05.269	100°17.662	2203
Or $_{M\ 1\text{-}3}$	79	87	0.91	As, Oi	04.06.12	Dry	Ma	21°04.116	100°16.107	2166
Or $_{Sf\ 1\text{-}1}$	64	72	0.89	Or	30.05.12	Dry	SF	21°38.470	101°03.437	1894
Or $_{Sf\ 1\text{-}2}$	73	87	0.84	Or	30.05.12	Dry	SF	21°40.223	101°02.681	1877
Or $_{Sf\ 1\text{-}3}$	60	48	1.25	Mamilaria sp.	30.05.12	Dry	SF	21°40.198	101°02.730	1879
Or $_{M\ 2\text{-}1}$	66	68	0.97		10.10.12	Rainy	Ma	21°05.192	100°17.715	2176
Or $_{M\ 2\text{-}2}$	83	69	1.20		10.10.12	Rainy	Ma	21°04.075	100°16.055	2176
Or $_{M\ 2\text{-}3}$	94	106	0.89		10.10.12	Rainy	Ma	21°04.062	100°16.048	2181
Or $_{Sf\ 2\text{-}1}$	82	98	0.84		12.10.12	Rainy	SF	21°10.202	101°02.652	1877
Or $_{Sf\ 2\text{-}2}$	59	74	0.80		12.10.12	Rainy	SF	21°40.194	101°02.754	1880
Or $_{Sf\ 2\text{-}3}$	65	68	0.96		12.10.12	Rainy	SF	21°38.526	101°03.225	1888

As: *Agave salmiana*
Oi: *Opuntia imbricata*

Table S2. Kruskal-Wallis test of the average relative abundance of bacterial/archaeal classes and fungal orders associated with *M. geometrizans* and *O. robusta* among the six plant compartments investigated. Significant differences ($P \leq 0.05$) are highlighted in green.

**16S rRNA Bacterial and Archaeal classes**				
Data	Degrees freedom (df)	Number of samples	X^2	*P*
Acidobacteria	5	47	28.628	2.742e-05
Actinobacteria	5	47	24.084	0.0002
Alphaproteobacteria	5	47	32.104	5.667e-06
Bacilli	5	47	19.300	0.0016
Betaproteobacteria	5	47	8.139	0.1487
Chloracidobacteria	5	47	30.401	1.229e-05
Deltaproteobacteria	5	47	31.913	6.18e-06
Gammaproteobacteria	5	47	23.380	0.0002
Gemmatimonadetes	5	47	34.690	1.735e-06
Oscillatoriophycideae	5	47	34.820	1.634e-06
Solibacteres	5	47	34.123	2.25e-06
Spartobacteria	5	47	30.157	1.373e-05
Sphingobacteria	5	47	27.466	4.627e-05
Thaumarchaeota	5	47	38.83	2.57e-07
**ITS2 Fungal orders**				
Data	Degrees freedom (df)	Number of samples	X^2	*P*
Agaricales	5	47	16.977	0.0045
Capnodiales	5	47	30.838	1.008e-05
Chaetothyriales	5	47	27.191	5.234e-05
Coniochaetales	5	47	27.700	4.165e-05
Diaporthales	5	47	31.306	8.15e-06
Dothideales	5	47	25.2042	0.0001
Eurotiales	5	47	16.613	0.005294
Ascomycota	5	47	7.791	0.1681
Helotiales	5	47	28.403	3.034e-05
Hypocreales	5	47	21.8459	0.0005
Hysteriales	5	47	15.339	0.0090
Leotiales	5	47	14.655	0.0119
Pezizales	5	47	22.093	0.0005
Pleosporales	5	47	21.412	0.0006
Sordariales	5	47	22.817	0.0003
Xylariales	5	47	5.560	0.3513

Table S3. Kruskal-Wallis test of the average relative abundance of the bacterial/archaeal classes and fungal orders associated with *M. geometrizans* and *O. robusta* among the four plant-associated compartments (rhizosphere, root endosphere, stem endosphere and phyllosphere). Significant differences ($P \leq 0.05$) are highlighted in green.

**16S rRNA<br>Bacterial and Archaeal classes**				
Data	Degrees freedom (df)	Number of samples	X^2	*P*
Acidobacteria	3	31	19.802	0.0001
Actinobacteria	3	31	17.021	0.0006
Alphaproteobacteria	3	31	20.835	0.0001
Bacilli	3	31	11.049	0.0114
Betaproteobacteria	3	31	4.692	0.1958
Chloracidobacteria	3	31	5.966	0.1132
Deltaproteobacteria	3	31	12.880	0.0049
Gammaproteobacteria	3	31	1.668	0.6441
Gemmatimonadetes	3	31	18.315	0.0003
Oscillatoriophycideae	3	31	19.429	0.0002
Solibacteres	3	31	13.957	0.0029
Spartobacteria	3	31	4.9757	0.1736
Sphingobacteria	3	31	19.951	0.0001
Thaumarchaeota	3	31	18.867	0.0003
**ITS2<br>Fungal orders**				
Data	Degrees freedom (df)	Number of samples	X^2	*P*
Agaricales	3	31	10.790	0.0129
Capnodiales	3	31	18.523	0.0003
Chaetothyriales	3	31	20.045	0.0001
Coniochaetales	3	31	17.502	0.0005
Diaporthales	3	31	20.488	0.0001
Dothideales	3	31	17.304	0.0006
Eurotiales	3	31	9.502	0.0233
Ascomycota	3	31	2.1193	0.548
Helotiales	3	31	17.474	0.0005
Hypocreales	3	31	15.634	0.0013
Hysteriales	3	31	10.045	0.0181
Leotiales	3	31	5.138	0.1619
Pezizales	3	31	5.555	0.1354
Pleosporales	3	31	12.875	0.0049
Sordariales	3	31	16.154	0.0010
Xylariales	3	31	3.714	0.294

Table S4. Analysis of similarity (AnoSim) using the Bray-Curtis dissimilarity matrix across all Cacti samples.

	Factor	16S rRNA Bacteria and Archaea		ITS2 Fungi	
		**AnoSim R**	***P***	**AnoSim R**	***P***
**Above-ground**	[a]Plant compartment$_{2,16/15,999}$	0.9782	0.001	0.9978	0.001
	[b]Plant compartment$_2$ by Species$_{2,8/7,999}$	0.9782	0.001	0.9978	0.001
	[b]Plant compartment$_2$ by Season$_{2,8/7,999}$	0.9782	0.002	0.9978	0.001
	[b]Plant compartment$_2$ by Site$_{2,8/7,999}$	0.9782	0.001	0.9978	0.001
	[b]Species$_2$ by Plant compartment$_{2,8/7,999}$	-0.02623	0.050	-0.02551	0.033
**Below-ground**	[a]Plant compartment$_{2,15/16,999}$	0.7063	0.002	0.4900	0.001
	[b]Plant compartment$_2$ by Species$_{2,7/8,999}$	0.7063	0.001	0.4900	0.002
	[b]Plant compartment$_2$ by Season$_{2,7/8,999}$	0.7063	0.001	0.4900	0.002
	[b]Plant compartment$_2$ by Site$_{2,7/8,999}$	0.7063	0.001	0.4900	0.002
	[b]Species$_2$ by Plant compartment$_{2,8,999}$			0.0820	0.017
	[b]Site$_2$ by Plant compartment$_{2,8,999}$			0.1066	0.013
**Episphere**	[a]Plant compartment$_{2,16,999}$	0.5742	0.001	0.9944	0.001
**Endosphere**	[a]Plant compartment$_{2,15,999}$	0.8061	0.001	0.8214	0.001
	[a]Species$_{2,15,999}$	0.2128	0.044		
**Soils**	[a]Site$_{2,16,999}$	0.2483	0.036		

a. Subscript numbers separated by comas indicate for each factor: the number of levels, number of total replicates and permutations employed in each AnoSim test.
b. Subscript numbers indicate for the first factor the number of levels and for the second factor, the subscript numbers separated by comas: the number of levels, number of total replicates and permutations employed in each AnoSim test. In particular cases, the diagonal separates the bacteria/archaea (left) and fungi (right) data.

Table S5. Kruskal-Wallis test of the estimated Shannon diversity index by factor in the bacterial/archaeal and fungal communities associated with *M. geometrizans* and *O. robusta*. Significant differences ($P \leq 0.05$) are highlighted in green.

**16S rRNA Bacterial and Archaeal community**					
Data	Factor	Degrees freedom (df)	Number of samples	Shannon index	
				X^2	P
Total	Plant compartment	5	47	34.906	1.57e-06
	Species	1		1.272	0.2594
	Season	1		0.011	0.9153
	Site	1		0.004	0.9491
M. geometrizans	Plant compartment	5	23	17.588	0.0035
	Season	1		0.378	0.5383
	Site	1		0.242	0.6225
O. robusta	Plant compartment	5	24	18.490	0.0023
	Season	1		0.270	0.6033
	Site	1		0.163	0.6861
**ITS2 Fungal community**					
Data	Factor	Degrees freedom (df)	Number of samples	Shannon index	
				X^2	P
Total	Plant compartment	5	47	27.526	4.50e-05
	Species	1		0.261	0.6095
	Season	1		2.413	0.1203
	Site	1		1.043	0.3070
M. geometrizans	Plant compartment	5	24	17.588	0.0030
	Season	1		0.379	0.5383
	Site	1		0.242	0.6225
O. robusta	Plant compartment	5	23	12.865	0.0246
	Season	1		0.458	0.4984
	Site	1		0.136	0.7119

Table S6. Estimated Shannon alpha diversity index in the bacterial/archaeal and fungal communities associated with *M. geometrizans* and *O. robusta*.

Plant compartment	16S rRNA Bacteria and Archaea		ITS2 Fungi	
	M. geometrizans[a]	***O. robusta***[b]	***M. geometrizans***[a]	***O. robusta***[b]
**Phyllosphere**	4.385 ± 0.262	3.74 ± 1.098	3.055 ± 0.26	2.176 ± 0.715
**Stem endosphere**	2.654 ± 0.526	2.139 ± 0.78	1.905 ± 0.447	2.173 ± 1.035
**Root endosphere**	3.227 ± 1.239	3.387 ± 0.896	2.191 ± 1.284	2.399 ± 1.187
**Rhizosphere**	3.004 ± 1.761	1.919 ± 1.01	3.803 ± 0.114	3.393 ± 0.257
**Root-zone soil**	4.948 ± 0.149	4.806 ± 0.137	3.936 ± 0.062	3.647 ± 0.67
**Bulk soil**	4.908 ± 0.158	4.879 ± 0.175	4.099 ± 0.175	3.723 ± 0.403

The values in the table represent the mean of the 4 replicates and its standard deviation in each case.
a. Statistically significant difference among plant compartments associated with *M. geometrizans* after Kruskal-Wallis test for Shannon index in Bacteria and Archaea ($H_{5,\,23}$ = 17.588, P= 0.0035) and Fungi ($H_{5,\,24}$ = 15.480, P= 0.0084).
b. Statistically significant difference among plant compartments associated with *O. robusta* after Kruskal-Wallis test for Shannon index in Bacteria and Archaea ($H_{5,\,24}$ = 18.490, P= 0.0023) and Fungi ($H_{5,\,23}$ = 12.865, P= 0.0246).

Table S7. Kruskal-Wallis test of the relative abundance of shared bacterial/archaeal classes and fungal orders among the two Cacti species in each plant compartment. Only classes and orders with significant differences ($P \leq 0.05$) are displayed.

**16S rRNA Bacteria and Archaea classes**					
Plant compartment	Data	Degrees freedom (df)	Number of samples	X^2	P
Phyllosphere	*Alphaproteobacteria*	3	20	4.084	0.0433
Stem endosphere	*Actinobacteria*	3	20	4.083	0.0433
Rhizosphere	*Gammaproteobacteria*	3	20	5.333	0.0209

**ITS2 Fungal orders**					
Plant compartment	Data	Degrees freedom (df)	Number of samples	X^2	P
Rhizosphere	*Chaetothyriales*	3	20	4.083	0.0433
	Sordariales			4.083	0.0433
Root endosphere	*Ascomycota*	3	20	5.333	0.0209
	Sordariales			4.083	0.0433

Table S8. Pareto analyses of microbial members associated with the stem endosphere of sympatric *M. geometrizans* and *O. robusta*. Cells highlighted in green correspond to species-specific taxa.

OTU	**Lineage** Kingdom|Phylum|Class|Order|Family|Genus|Species	**% Relative abundance**	**% Relative frequency**
**16S rRNA Bacteria and Archaea**			
M. geometrizans			
19[abd]	Bacteria|Firmicutes|Bacilli|Bacillales|Bacillaceae|Bacillus	31.21	100
237[ab]	Bacteria|Bacteroidetes|Sphingobacteria|Sphingobacteriales|SphingobacterialesOR|SphingobacterialesOR	12.68	100
174[ab]	Bacteria|Bacteroidetes|Sphingobacteria|Sphingobacteriales|SphingobacterialesOR|SphingobacterialesOR	7.37	100
4[abcd]	Bacteria|Proteobacteria|Gammaproteobacteria|Enterobacteriales|Enterobacteriaceae|Leclercia	6.86	100
20[abc]	Bacteria|Proteobacteria|Gammaproteobacteria|Pseudomonadales|Moraxellaceae|Acinetobacter	5.27	100
10[ab]	Bacteria|Proteobacteria|Gammaproteobacteria|Pseudomonadales|Moraxellaceae|Acinetobacter	4.01	50
99[abcd]	Bacteria|Firmicutes|Bacilli|Bacillales|Staphylococcaceae|Staphylococcus	3.50	100
371[abc]	Bacteria|Proteobacteria|Gammaproteobacteria|Enterobacteriales|Enterobacteriaceae|Escherichia	2.49	75
73[ab]	Bacteria|Firmicutes|Bacilli|Bacillales|BacillalesOR|BacillalesOR	2.31	25
24[ab]	Bacteria|Proteobacteria|Betaproteobacteria|Burkholderiales|Burkholderiaceae|Burkholderia	1.90	100
372[ab]	Bacteria|Bacteroidetes|Sphingobacteria|Sphingobacteriales|SphingobacterialesOR|SphingobacterialesOR	1.57	100
657[a]	Bacteria|Cyanobacteria|4C0d_2|mle1_12|mle1_12OR|mle1_12OR	1.52	100
O. robusta			
19[abd]	Bacteria|Firmicutes|Bacilli|Bacillales|Bacillaceae|Bacillus	29.3	100
237[ab]	Bacteria|Bacteroidetes|Sphingobacteria|Sphingobacteriales|SphingobacterialesOR|SphingobacterialesOR	19.3	100
12[abcd]	Bacteria|Proteobacteria|Gammaproteobacteria|Xanthomonadales|Xanthomonadaceae|Stenotrophomonas	11.14	100
174[ab]	Bacteria|Bacteroidetes|Sphingobacteria|Sphingobacteriales|SphingobacterialesOR|SphingobacterialesOR	9.8	100
371[abc]	Bacteria|Proteobacteria|Gammaproteobacteria|Enterobacteriales|Enterobacteriaceae|Escherichia	4.7	100
24[ab]	Bacteria|Proteobacteria|Betaproteobacteria|Burkholderiales|Burkholderiaceae|Burkholderia	3.56	100
372[ab]	Bacteria|Bacteroidetes|Sphingobacteria|Sphingobacteriales|SphingobacterialesOR|SphingobacterialesOR	2.65	100
**ITS2 Fungi**			
M. geometrizans			
339[b]	Fungi|Basidiomycota|Agaricomycetes|Agaricales|Marasmiaceae|Henningsomyces	32.94	25
5[abc]	Fungi|Ascomycota|Dothideomycetes|Pleosporales|Pleosporaceae|Alternaria	15.4	50
1575[ab]	Fungi|Ascomycota|Dothideomycetes	12.04	100
1316[ab]	Fungi|Basidiomycota|Agaricomycetes|Agaricales	7.09	100
2[abc]	Fungi|Ascomycota|Dothideomycetes|Capnodiales|Davidiellaceae|Cladosporium	6.56	50
320[ab]	Fungi|Ascomycota|Sordariomycetes|Hypocreales|Cordycipitaceae|Beauveria	3.8	100
50[ab]	Fungi|Basidiomycota|Agaricomycetes	2.28	25
O. robusta			
103[a]	Fungi|Ascomycota|Dothideomycetes|Pleosporales|Pleosporaceae|Prathoda	70.77	66.67
320[ab]	Fungi|Ascomycota|Sordariomycetes|Hypocreales|Cordycipitaceae|Beauveria	8.03	66.67
2[abc]	Fungi|Ascomycota|Dothideomycetes|Capnodiales|Davidiellaceae|Cladosporium	3.65	100

a. OTUs present in the phyllosphere in the two Cacti species.
b. OTUs present in the root endosphere in the two Cacti species.
c. OTUs present in the dry core observed in *A. tequilana, A. saliana* and *A. deserti* reported in Coleman-Derr *et al.*, (2016).
d. OTUs with 98% homology with respect to isolated strains from the two Cacti species and in the process of genome sequencing.

Table S9. Quantification of IAA production and phosphate solubilization of the seed-borne isolated strains associated with *M. geometrizans* and *O. robusta*.

Strain	Phosphate solubilization (µgPO4/ml)[a]	IAA production (µgIAA/ml)[b]
B. pumilus L14	6.39±1.19	2.09±0.04
P. psychrodurans L11	59.42±5.03**	2.57±0.03
B. pumilus L1	58.55±2.25**	2.07±0.02
P. psychrodurans L4	63.08±4.08**	2.04±0.03
P. psychrodurans L5	29.4±5.95	20.5±0.12***
P.psychrodurans L6	63.36±5.02**	2.09±0.05
P. psychrodurans L7	58.65±2.52**	2.65±0.05
P.psychrodurans L8	22.81±7.62	2.17±0.08
P. psychrodurans L9	56.43±0.44**	2.85±0.18
P.psychrodurans L2	55.68±1.57**	1.99±0.01
P. psychrodurans L10	53.36±1.28**	2.61±0.03
S.hominis L12	83.5±2.78***	2.31±0.27
P. psychrodurans L13	53.1±2.13**	2.49±0.06
B. pumilus L3	51.45±2.62**	2.06±0.02
A. terreus L15	57.82±8.4**	16.82±0.15**
L. adecarboxylata L16	29.4±5.95	15.02±0.46**
N. prasina L17	59.18±9.39**	1.8±0.06

a. Statistically significant difference between isolated seed-borne strains associated with *M. geometrizans* and *O. robusta* after Kruskal-Wallis test for quantification of phosphate solubilization ($H_{16, 51}$ = 42.50, P= 0.0003).
b. Statistically significant difference between isolated seed-borne strains associated with *M. geometrizans* and *O. robusta* after Kruskal-Wallis test for quantification of IAA production ($H_{16, 51}$ = 47.20, P= 6.33e-^{05}).
Asterisk (** and ***) represent medium and high production values, respectively.
